# Structure of HEPES-Reduced δ‑MnO_2_ Nanosheets

**DOI:** 10.1021/acs.chemmater.5c03046

**Published:** 2026-04-15

**Authors:** Alain Manceau, Yan Li, Jianlin Liao, Valérie Magnin, Lorenzo Spadini, Catherine Dejoie, Olivier Mathon, Anne-Claire Gaillot

**Affiliations:** † European Synchrotron Radiation Facility (ESRF), 38043 Grenoble, France; ‡ Université Grenoble Alpes, CNRS, ISTerre, 38000 Grenoble, France; § Université Grenoble Alpes, CNRS, Institut des Géosciences de l’Environnement, 38000 Grenoble, France; ∥ Nantes Université, CNRS, Institut des Matériaux de Nantes Jean Rouxel, IMN, 44000 Nantes, France

## Abstract

The reduction of tetravalent manganese (Mn­(IV)) to trivalent manganese (Mn­(III)) by HEPES Good’s buffer is often used to modify the reactivity of δ-MnO_2_ and to distinguish between the Mn­(III) and Mn­(IV) oxidants in redox reactions. However, the structure of HEPES-reacted δ-MnO_2_ has remained elusive, hindering a detailed understanding of interfacial electron transfer between adsorbed species and structural Mn. Here, we characterized the structure of δ-MnO_2_ reacted with HEPES at pH 6 and 8 under low and high NaCl ionic strength, using chemical analysis, high-energy X-ray diffraction, pair distribution function (PDF), extended X-ray absorption fine structure (EXAFS) spectroscopy, and high-resolution transmission electron microscopy (HRTEM) coupled with selected area electron diffraction (SAED). The average Mn oxidation state (AMOS) decreases from 3.92−3.87 to 3.71–3.59 after HEPES addition, depending on pH and ionic strength. HEPES-reacted δ-MnO_2_ has a distinctly different structure at low and high ionic strength. At low ionic strength, the δ-MnO_2_^HE^ crystallites are 3–6 nm across, and the MnO_2_ layers have approximately 23% vacant sites capped with mainly Mn­(III) and some Mn­(II). At high ionic strength and pH 8, δ-MnO_2_^HE^ contains large crystals, several hundred nanometers across, made up of crystallographically oriented nanodomains. Most SAED patterns show streaks along the [100]* direction, indicating a high degree of disorder in the close packing of the anionic sheets, in the Na position within the interlayer, and in the Mn­(IV)–Mn­(III) distribution within the layer. Some nanodiffraction patterns show distinct superstructure reflections along the streaks with A* = 3a*, as seen in well-crystallized triclinic birnessite, and A* = 6a*. High-ionic-strength δ-MnO_2_^HE^ has no interlayer Mn­(III), and the Na­(I) ions, along with the layer Mn­(III) and Mn­(IV) cations, are semiordered at the short- to medium-range scales and essentially disordered over longer distances. Identifying the two distinct structures of HEPES-reacted δ-MnO_2_ clarifies structural ambiguities reported in the literature and provides a solid foundation for exploring its redox reactivity and electrochemical performance.

## Graphical abstract

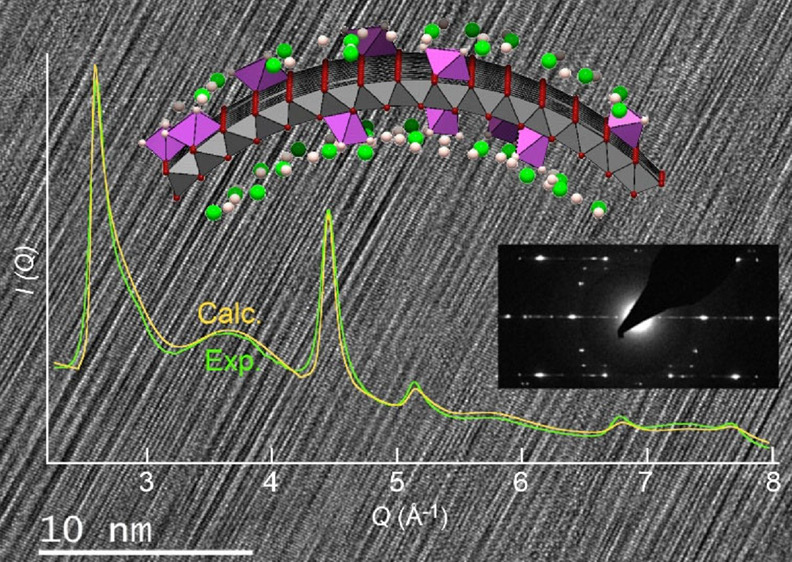

## 1 Introduction

Since its initial description in 1940 by Betekhtin,^1^ the natural layered manganate dioxide vernadite, and its synthetic counterpart δ-MnO_2_,^2^ have consistently attracted interest in environmental, chemical, and materials science. It is recognized as the most abundant and reactive Mn oxide at the Earth’s surface^3–8^ and marine deposits,^9–12^ where it critically influences the environmental fate and geochemical cycling of trace metals,^13–39^ and drives photocatalytic geochemical reactions.^40–43^ A very basic form of δ-MnO_2_ (cubane CaMn_4_ cluster) constitutes the core of the electron transfer process in Photosystem II.^44,45^ Its electrochemical and catalytic properties, combined with high sorption capacity, are used for energy storage and production (e.g., artificial photosynthesis, heat storage), in the chemical industry (e.g., CO and H_2_O oxidation, Fenton reaction, photothermal catalysis),^43–56^ and in wastewater treatment.^57^

The chemical reactivity of δ-MnO_2_ is due to its low dimensionality (2D), nanoparticulate size, defective structure, and nonstoichiometry.^58^ The δ-MnO_2_ flakes are typically 20–70 nm wide and contain discrete nanocrystals of 3–4 nm (Figures 1 and S1). The flakes are made up of 3–6 MnO_2_ layers, which are disorderly stacked at the particle scale. The MnO_2_ layers carry a negative charge because of varying amounts of Mn­(IV) vacancies and Mn­(III) for Mn­(IV) substitutions. Exchangeable alkali and alkaline earth metal ions, along with more strongly bonded cations, are adsorbed in the interlayer to neutralize this negative charge (Figure 2). The defective structure of natural δ-MnO_2_ (i.e., vernadite) has been described in a single study on the biomineralization of Zn in grass roots.^37^ Aside from this work, all chemical and structural investigations have focused on δ-MnO_2_ synthesized either chemically^59–83^ or biologically by bacteria and fungi.^84–96^ An example of structural formula,^58^ determined at pH 6 for a chemical δ-MnO_2_ (dBi6) synthesized after the “redox” method of Villalobos et al., is^87^

$$(H_{0.16}^{+}{\mathrm{Na}}_{0.18}^{+}(H_{2}O{)}_{0.3}{\mathrm{Mn}}_{0.15}^{3+}(H_{2}O{)}_{0.45}{)}_{\mathrm{IL}}({\mathrm{Mn}}_{0.72}^{4+}{\mathrm{Mn}}_{0.11}^{3+}{□}_{0.17}{)}_{L}O_{2}$$ 1

L and IL represent the layer and interlayer composition, respectively. The negative charge of the layer, caused by Mn­(III) replacing Mn­(IV) and the presence of octahedral vacancies (□) is balanced by Na­(I) and Mn­(III) cations in the interlayer. The average Mn oxidation state (AMOS) is 3.73.

**Figure 1:**
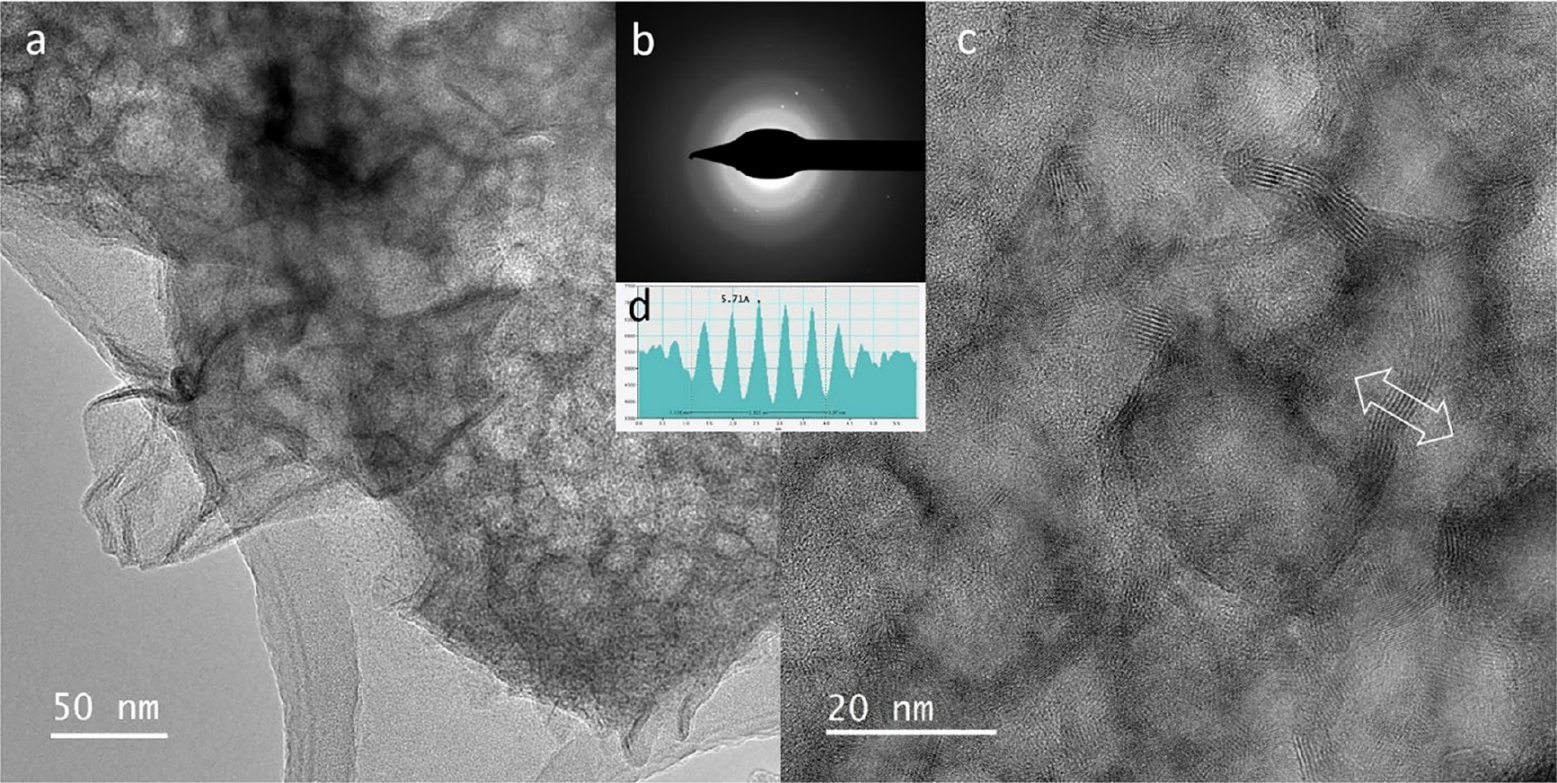
Transmission electron images and diffraction of δ-MnO_2_. (a) An aggregate of crumpled nanosheets. In the lower left, there is a 50 nm platelet with curved and rolled-up edges. (b) SAED pattern of a low-crystallinity particle (diffuse rings) and some nanocrystals (diffraction spots). (c) High-resolution image of crystallized nanodomains in the *ab* plane and platelets viewed on the edges along the *c* direction. (d) Intensity profile across the platelet marked with a double arrow. The *d*-spacing between the MnO_2_ layers is 5.7 Å due to layer collapse caused by dehydration in vacuum. The birnessite structure is stable under vacuum.^97^

**Figure 2:**
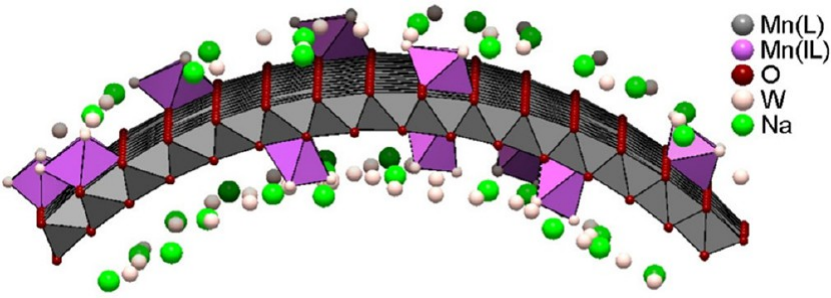
Structure of bent δ-MnO_2_ layer. After ref 58.

The HEPES buffer (4-(2-hydroxyethyl)-1-piperazineethanesulfonic acid, C_8_H_18_N_2_O_4_S) is commonly used in δ-MnO_2_ synthesis to maintain pH at 7.5. Elzinga and Kustka^98^ found in 2015 that Mn­(IV) was unintentionally reduced to Mn­(III) by HEPES, and they observed a small change in the X-ray diffraction (XRD) pattern after 2 days of reaction in a glovebox at pH 7.5 and 0.1 M NaCl ionic strength compared to unreacted δ-MnO_2_. The XRD pattern of the initially synthesized δ-MnO_2_ is largely featureless.^99^ The 001 and 002 basal reflections are weak and broad due to the limited number of layers in the diffracting crystallites, and the *hkl* reflections are replaced by so-called broad *hk* bands at 2.46 Å (20,11) and 1.42 Å (02,31) because of the turbostratic layer stacking.^58^ Except for one biogenic δ-MnO_2_ synthesized by *Bacillus* sp. strain SG-14,^100^ the *d*-spacing ratio of the 20,11 and 02,31 reflections is always $a=b\sqrt{3}$ , which indicates a hexagonal layer symmetry (Figure 3a). As an aside, we note that the 20,11 and 02,31 *hk* indices in a C-centered orthogonal layer cell are equivalent to the 100 and 110 *hkl* indices in a hexagonal indexing of the layer cell, as sometimes reported in the literature.^58,99^ Elzinga and Kustka^98^ observed sharper 00*l* reflections with HEPES, indicating more layers stacked coherently along the *c*-axis, and a shoulder on the 20,11 and 02,31 reflections, suggesting a change from hexagonal to orthogonal layer symmetry ( $a\neq b\sqrt{3}$ , Figure 3a).

**Figure 3:**
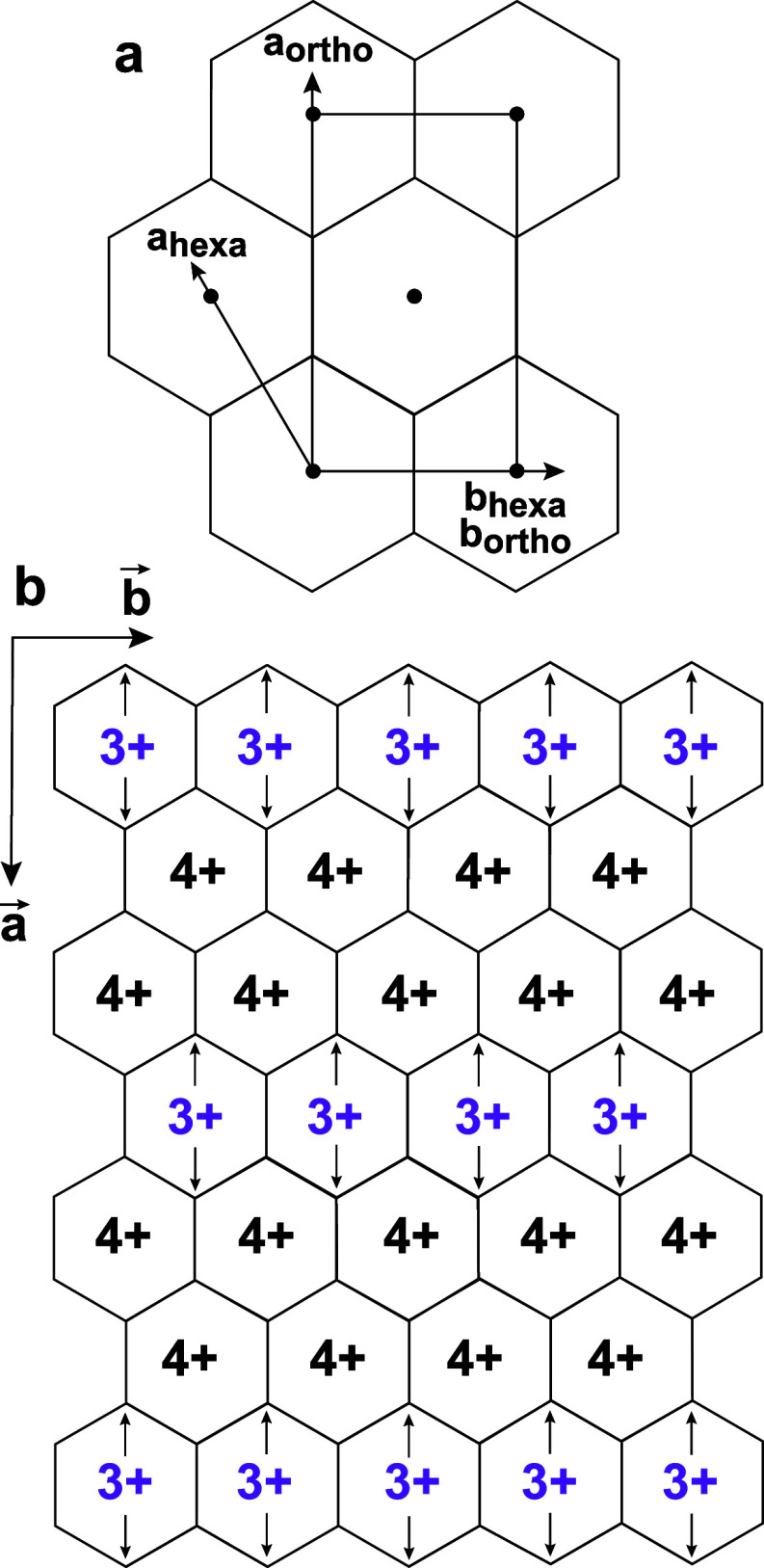
(a) Representation of the hexagonal and orthogonal cells. If the symmetry is hexagonal, $a_{\mathrm{ortho}}=b_{\mathrm{ortho}}\sqrt{3}$ (b) Distribution of the Mn­(III) and Mn­(IV) cations in monoclinic/triclinic birnessite (TcBi).^99,101^ Arrows indicate the direction of the elongated Mn–O distances caused by the Jahn–Teller distortion.

Orthogonal layer symmetry is observed in monoclinic/triclinic birnessite (TcBi, nominal composition Na_0.31_^1+^(Mn_0.69_^4+^Mn_0.31_^3+^)­O_2_ · 0.40H_2_O). It results from the long-range ordering of the Mn cations in Mn­(IV)- and Mn­(III)-rich rows along the *b* axis, which alternate with an *A* = 3*a* supercell periodicity (−Mn­(III)–Mn­(IV)–Mn­(IV)–Mn­(III)−) (Figure 3b).^99,101^ The AMOS value of TcBi is 3.69. Elzinga and Kustka^98^ linked the increased ordering along the *c* axis of HEPES-reacted δ-MnO_2_ (referred to as δ-MnO_2_^HE^) to the replacement of Na by Mn­(II)/Mn­(III) cations in the interlayer. The reduction in layer symmetry suggested the incorporation of Mn­(III) into vacancy sites and a partial TcBi-like ordering of the Mn­(IV) and Mn­(III) cations within the MnO_2_ layer.

Independent of Elzinga and Kustka,^98^ Simanova et al.^102^ characterized, also in 2015, δ-MnO_2_^HE^ reacted for 2 days at pH 6.6 and 10 mM NaCl ionic strength using extended X-ray absorption fine structure (EXAFS) spectroscopy and pair distribution function (PDF). Neither technique showed evidence of departure from hexagonal symmetry, contrasting with Elzinga and Kustka’s XRD findings.^98^ The orthogonal layer symmetry of TcBi is visually seen as a double antinode pattern of the EXAFS photoelectron wave at *k* = 7.9 Å^–1^ (Figure 4a).^15–17^ This EXAFS feature, used to differentiate between hexagonal birnessite (HBi) and TcBi, was absent in the publication of Simanova et al.^102^ However, potentiometric titration indicated that HEPES reacted with Mn­(IV), as the AMOS value was 3.65 ± 0.05 v.u., similar to TcBi. To reconcile the hexagonal layer symmetry, the authors suggested that the surplus Mn­(III) not sitting on vacancies in the interlayer (eq 1) probably resided at the layer edges. This interpretation was further supported by Sun et al.,^103^ who synthesized δ-MnO_2_^HE^ at pH 7 for 2 days and characterized it using chemical analysis, XRD, and X-ray photoelectron spectroscopy (XPS). The layer symmetry observed using XRD by Sun et al.^103^ was also hexagonal, and the Mn­(II):Mn­(III):Mn­(IV) ratio was 2:34:64 based on XPS and chemical analysis. Approximately 11% Mn­(III) was presumed to be located on vacancies, and 23% was thought to be on the layer edges.

**Figure 4:**
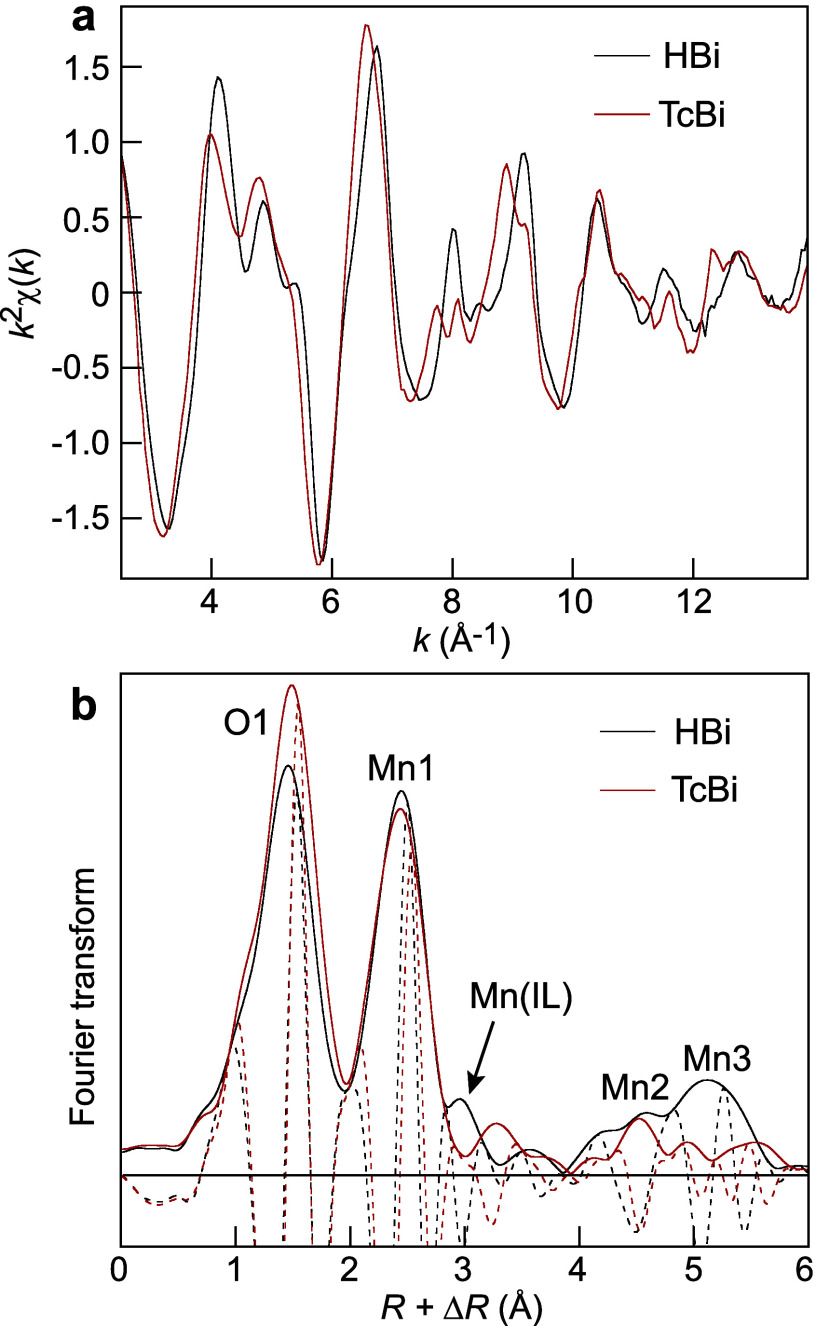
Mn K-edge EXAFS spectrum (a) and radial distribution function (RDF, Fourier transform) (b) of hexagonal (HBi) and triclinic (TcBi) birnessite. After ref 104.

While there is clear consensus from previous studies that care should be taken to prevent the undesired reduction of Mn­(IV) by HEPES buffer in the synthesis of δ-MnO_2_, Hausladen and Peña^59^ showed that biogenic δ-MnO_2_ is less susceptible to Mn reduction by HEPES than chemical δ-MnO_2_. However, HEPES’s reducing properties on chemical δ-MnO_2_ have important beneficial effects for studying the oxidation mechanism of redox elements.^105^ Wick et al.^62^ observed that Tl­(I) was less oxidized to Tl­(III) on δ-MnO_2_^HE^ than on δ-MnO_2_, which they attributed to the blocking of the redox-reactive vacancy sites^106,107^ by interlayer Mn­(III) in δ-MnO_2_^HE^. The hindrance of Mn­(III) to the oxidative uptake of Tl suggested that Mn­(IV) was the electron acceptor,^62^ which is supported by density functional theory (DFT).^108^

The main goal of this study is to determine the structure of δ-MnO_2_^HE^ through the combination of chemical analysis, high-energy XRD, PDF, EXAFS spectroscopy, high-resolution transmission electron microscopy (HRTEM), and selected area electron diffraction (SAED). A specific motivation is to clarify the structural location of Mn­(III), whether it is incorporated and orderly distributed inside the δ-MnO_2_^HE^ layer or located on the edge sites. This information is essential for advancing the understanding of the chemical reactivity of δ-MnO_2_^HE^ with broad applications in biogeochemistry and materials science. For this purpose, δ-MnO_2_ and δ-MnO_2_^HE^ were synthesized at pH 6 and 8 (δ-MnO_2_-6/8, δ-MnO_2_^HE^-6/8), and at low (<0.01 M NaCl) and high (0.6 M NaCl) ionic strengths (IS). The aging time was 3 days for δ-MnO_2_ and 7 days for δ-MnO_2_^HE^ to promote Mn­(IV)–Mn­(III) order. The pH selection was based on the stability range of HBi (pH ≤ 6) and TcBi (pH ≥ 8).^109^

## 2 Results and Discussion

### 2.1 Structure of δ-MnO_2_^HE^ at Low Ionic Strength

#### 2.1.1 Chemical Formula

Omitting protonated surface sites at crystal edges, the generic structural formula of δ-MnO_2_ is (H_*n*_^+^Na_*v*_^+^(H_2_O)_*w*_Mn_*x*_^2+/3+^(H_2_O)_3*x*_)_IL_(Mn_1–*y*–*z*_^4+^Mn_*y*_^3+^□_*z*_)_L_O_2_, where *w* is the amount of strongly sorbed water molecules and 3*x* is the amount of water molecules bonded to interlayer Mn atoms.^58^ The *w* and 3*x* values of δ-MnO_2_-6/8 and δ-MnO_2_^HE^-6/8 were obtained by measuring the weight loss percentage between 80 and 250 °C using thermogravimetric analysis (TGA) coupled with differential thermal analysis (DTA) (Figure S2).^97,104,110,111^ The structural water content is similar across the samples, ranging from 13.3 to 14.2% by mass. The Na/Mn atomic ratios measured by chemical analysis are 0.21 (pH 6) and 0.28 (pH 8) for δ-MnO_2_, and 0.03 (pH 6) and 0.05 (pH 8) for δ-MnO_2_^HE^. The AMOS values determined by pyrophosphate extraction of Mn­(III) and chemical analysis of Mn­(II) are 3.87–3.88 (pH 6 and 8) for δ-MnO_2_, and 3.59 (pH 6) and 3.66 (pH 8) for δ-MnO_2_^HE^ (Table S1). The lower AMOS values indicate reduction of Mn­(IV) to Mn­(III) (26–29%) and Mn­(II) (4–6%) by HEPES, confirming its strong reducing power. The chemical formulas derived from these analyses are reported in Table 1. The positive and negative charges are unbalanced because the distribution of the Mn cations between the layer and interlayer sites and the vacancy content are unknown. The objectives which follow are to determine (1) the structural formulas (i.e., *x* and *z* values) of δ-MnO_2_-6/8 and δ-MnO_2_^HE^-6/8, and (2) the structure of δ-MnO_2_^HE^-6/8, using long-range (XRD, SAED), medium-range (PDF, HRTEM), and short-range (EXAFS) structural probes.

**Table 1:** Chemical and Structural Formulae and AMOS of δ-MnO_2_-6/8 and δ-MnO_2_^HE^-6/8 at Low Ionic Strength

**sample**	**chemical formula** ^a^	**structural formula** ^b^	**AMOS**
δ-MnO_2_-6 – low IS	Na_0.21_^+^Mn_0.01_^2+^Mn_0.11_^3+^Mn_0.88_^4+^O_2_ · 0.75H_2_O	(Na_0.21_^+^(H_2_O)_0.46_^s^Mn_0.01_^2+^Mn_0.07_^3+^(H_2_O)_0.24_^b^)_IL_(Mn_0.86_^4+^Mn_0.04_^3+^□_0.10_)_L_O_2_	3.87
δ-MnO_2_-8 – low IS	Na_0.28_^+^Mn_0.01_^2+^Mn_0.11_^3+^Mn_0.88_^4+^O_2_ · 0.77H_2_O	(Na_0.29_^+^(H_2_O)_0.40_^s^Mn_0.01_^2+^Mn_0.10_^3+^(H_2_O)_0.33_^b^)_IL_(Mn_0.85_^4+^□_0.15_)_L_O_2_	3.88
δ-MnO_2_^HE^-6 – low IS	Na_0.03_^+^Mn_0.06_^2+^Mn_0.29_^3+^Mn_0.65_^4+^O_2_ · 0.72H_2_O	(H_0.38_^+^Na_0.03_^+^Mn_0.06_^2+^Mn_0.17_^3+^(H_2_O)_0.69_^b^)_IL_(Mn_0.65_^4+^Mn_0.12_^3+^□_0.23_)_L_O_2_	3.59
δ-MnO_2_^HE^-8 – low IS	Na_0.05_^+^Mn_0.04_^2+^Mn_0.26_^3+^Mn_0.70_^4+^O_2_ · 0.71H_2_O	(H_0.29_^+^Na_0.05_^+^Mn_0.04_^2+^Mn_0.19_^3+^(H_2_O)_0.69_^b^)_IL_(Mn_0.70_^4+^Mn_0.07_^3+^□_0.23_)_L_O_2_	3.66

a From chemical analysis (Table S1) and TGA.

b (H_2_O)^s^ are strongly sorbed water molecules, (H_2_O)^b^ are water molecules bonded to interlayer Mn. (H_2_O)^s^ and (H_2_O)^b^ were determined by TGA.

#### 2.1.2 High-Energy XRD

The diffraction patterns of the initial δ-MnO_2_-6/8 are similar and characteristic of turbostratic δ-MnO_2_ nanoparticles (Figure 5). The maxima of the 001 and 002 basal reflections occur at 7.7–8.1 Å and 3.9 Å, while the 20,11 and 02,31 *hk* reflections are observed at 2.43 and 1.41 Å (Figure 5a). The 2.43:1.41 ratio is close to $1.732=\sqrt{3}$ , in line with hexagonal layer symmetry (Figure 3a). The XRD patterns for low-IS δ-MnO_2_^HE^-6 and δ-MnO_2_^HE^-8 are also similar, differing from the δ-MnO_2_ patterns by a broad hump between 1.9 and 1.5 Å (Figures 5a and S3). Its intensity increases with the electronic density in the interlayer, primarily depending on the proportion of interlayer Mn atoms (Mn­(IL)), and secondarily on the density of vacancy sites (i.e., proportion of Mn­(L)).^58,70,89,111,112^

**Figure 5:**
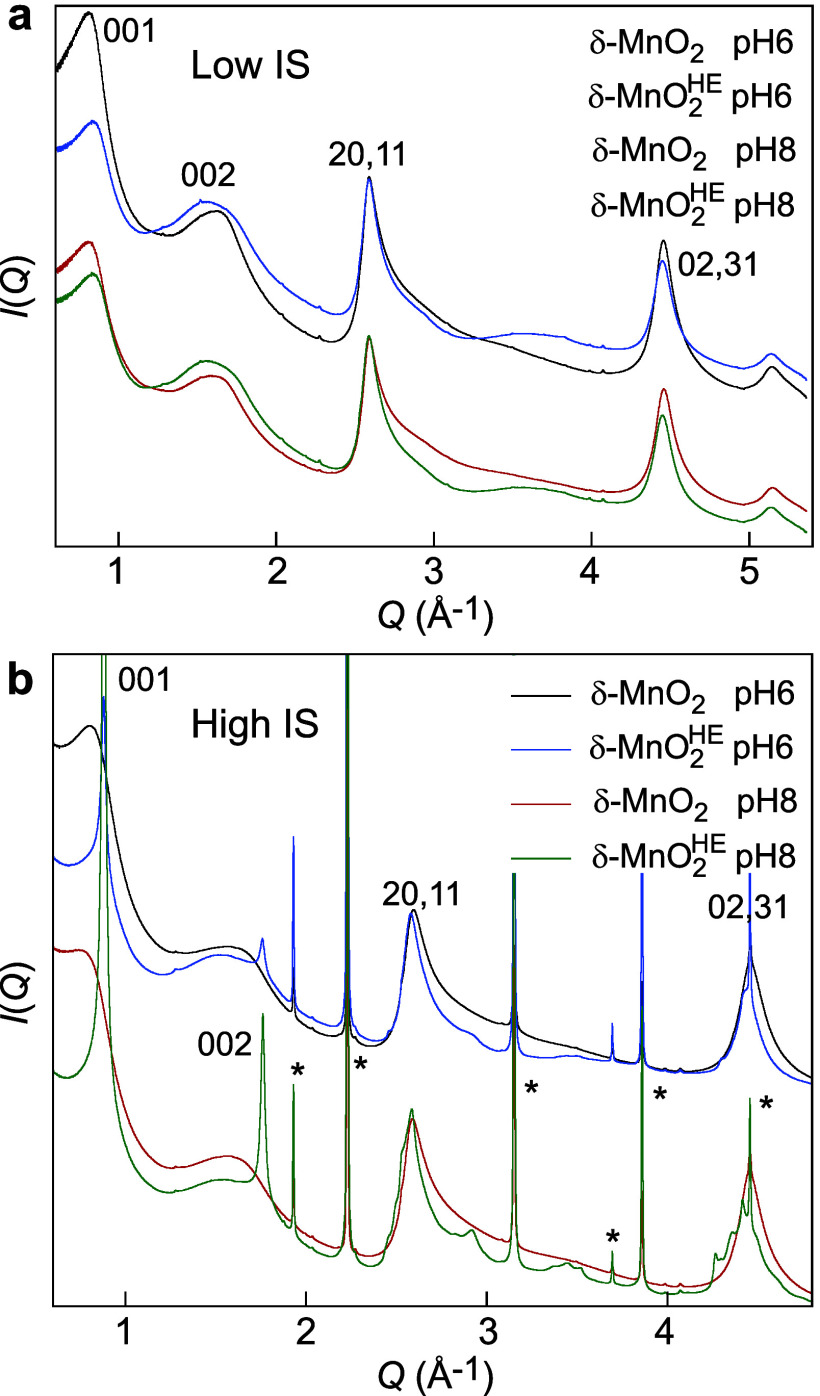
High-energy diffraction patterns of δ-MnO_2_-6/8 and δ-MnO_2_^HE^-6/8 at low (a) and high (b) IS. The sharp diffraction lines at high IS are from NaCl (*). *Q*(Å^−1^) = 2π/*d*(Å).

The structural formulas of δ-MnO_2_-6/8 and low-IS δ-MnO_2_^HE^-6/8 were determined by modeling the X-ray diffraction patterns in the 2.25 Å^–1^ ≤ *q* ≤ 5.0 Å^–1^ range (1.25 Å ≤ *d* ≤ 2.8 Å), which is where the X-ray scattering intensity is most sensitive to variations in the structure factor of phyllomanganate nanoparticles.^58^ The site occupancies of the Mn­(L) and Mn­(IL) positions were refined, while the Na/Mn ratios were fixed to values obtained by chemical analysis. The atomic coordinates of all atoms (O, Mn­(L), Mn­(IL), Na, H_2_O) were fixed to those previously determined for δ-MnO_2_.^58^ Best-model calculations are shown in Figure 6, with details on the structural and fit parameters provided in Tables S2 and S3. Although calculations were performed within the 2.25 Å^–1^ ≤ *q* ≤ 5.0 Å^–1^ interval, the theoretical and experimental XRD patterns are plotted up to *q* = 8.1 Å^–1^ in Figure 6 to demonstrate the validity of the physical approach. The agreement between data and calculations is reasonably good, and the optimal parameter values align with previous results on similar materials.^58,89,111,112^ The structural formulas are reported in Table 1.

**Figure 6:**
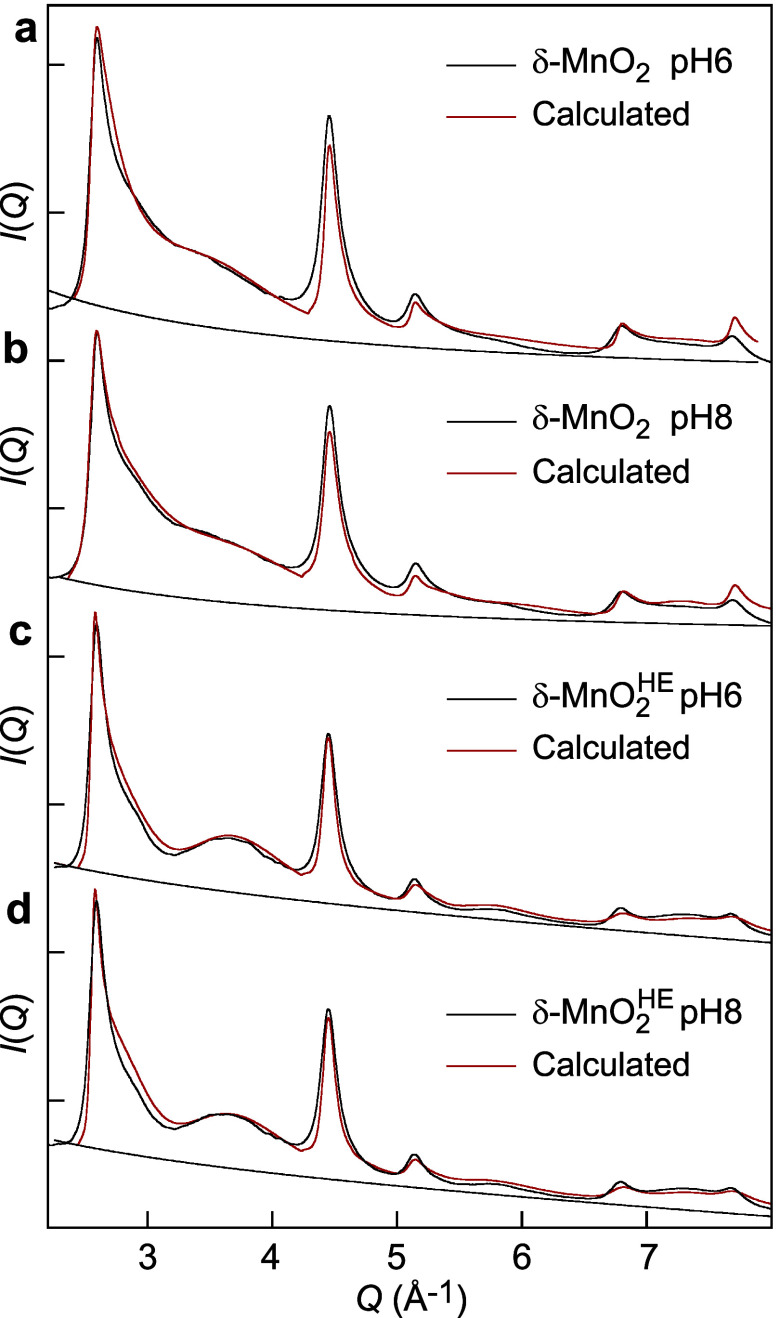
Simulation of the high-energy diffraction patterns of MnO_2_ and δ-MnO_2_^HE^ at low IS. (a) MnO_2_-6. (b) MnO_2_-8. (c) δ-MnO_2_^HE^-6. (d) δ-MnO_2_^HE^-8.

The main result and key difference from all known δ-MnO_2_ structures is the high Mn­(IL) content (17–19% Mn­(III) and 4–6% Mn­(II)) of the two δ-MnO_2_^HE^-6/8 at IS < 0.01, compared to δ-MnO_2_-6/8 (7–10% Mn­(III) and 1% Mn­(II)). To our knowledge, Mn­(IL) exceeds all reported values. The δ-MnO_2_^HE^-6/8 layers also have noticeably more Mn­(III) cations (7–12%) than the δ-MnO_2_-6/8 layers (0–4%). Thus, HEPES reduces Mn­(IV) to Mn­(III) and Mn­(II), which then migrate out of the layer into vacancy-capping sites. It should be noted that the Mn­(III) layer content cannot be directly inferred from the *a*,*b* unit-cell parameters, because the layer dimension depends on several factors, including the fraction of both Mn­(III) and vacancies, as well as the microstructure of the nanosheets. The Mn–Mn distances relax around vacancies, and the MnO_2_ nanosheets are not flat but curved (Figures 1 and 2), which modifies the apparent lattice parameters.^58^

#### 2.1.3 PDF

The validity of the XRD structure models was confirmed using high-energy X-ray total scattering (S­(*Q*)) combined with real-space PDF analysis. The Fourier transform of *Q*[S­( *Q* )-1] produces the PDF, or G­(*R*), which shows peaks at interatomic distances between all atomic pairs (Figure 7). The PDF of δ-MnO_2_ is dominated by the strongest Mn–O and Mn–Mn correlations at short- and intermediate-range distances.^51,113,58,72,102,114–117^ The peaks up to *R* = 7 Å represent atomic pairs within the layer and in the interlayer space. Correlations from atomic pairs in two consecutive MnO_2_ layers appear at greater distances.

**Figure 7:**
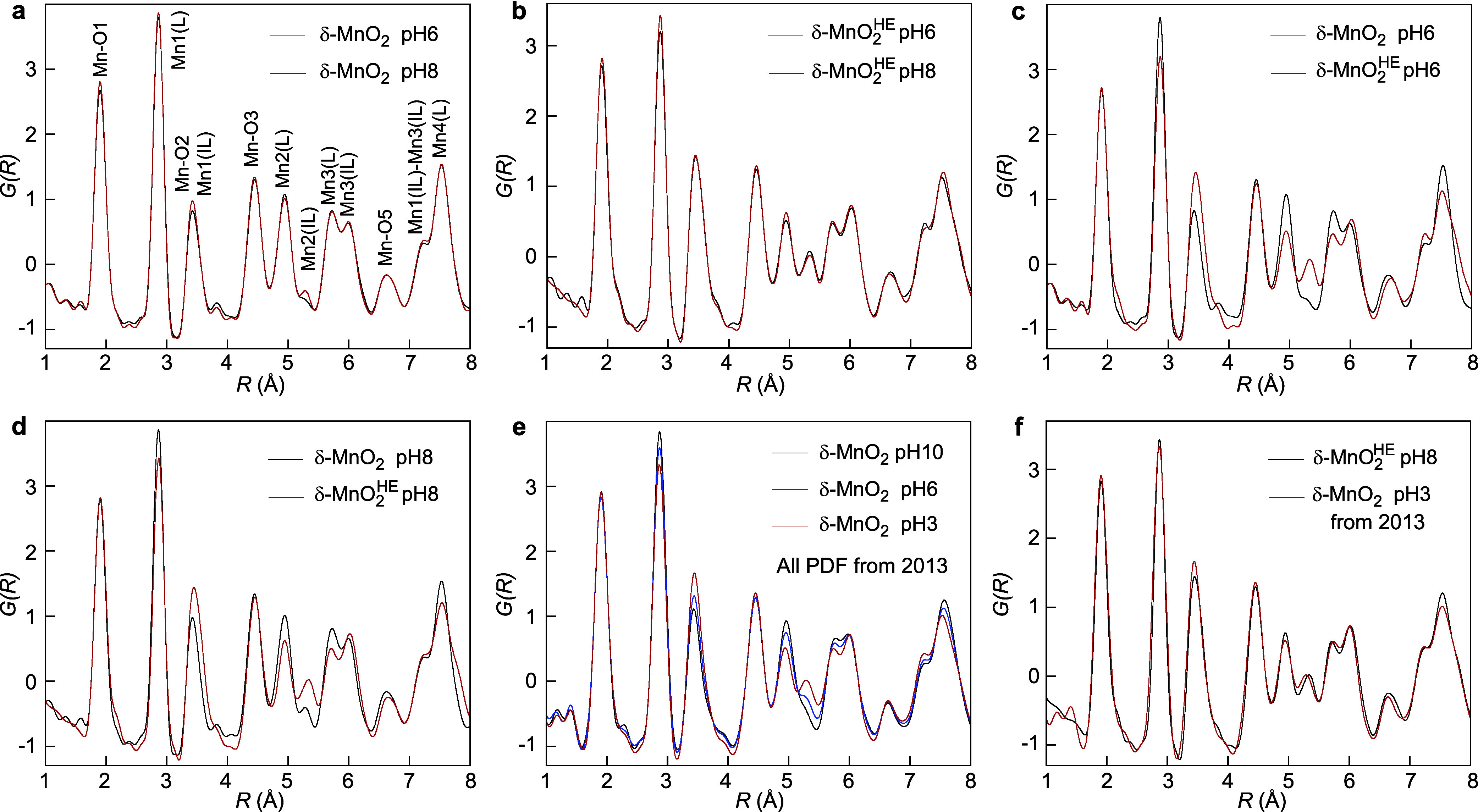
PDF of δ-MnO_2_ and δ-MnO_2_^HE^. (a) δ-MnO_2_-6/8 at low IS. (b) δ-MnO_2_^HE^-6/8 at low IS. (c) δ-MnO_2_-6 and δ-MnO_2_^HE^-6 at low IS. (d) δ-MnO_2_-8 and δ-MnO_2_^HE^-8 at low IS. (e) δ-MnO_2_ equilibrated at pH 3, 6, and 10 at IS = 0.01 M NaCl.^58^ (f) δ-MnO_2_-3 at IS = 0.01 M NaCl and δ-MnO_2_^HE^-8 at low IS (<0.01 M NaCl).

The first peak at ca. 1.90 Å corresponds to the Mn–O_1_ distance. The second peak at ca. 2.86 Å represents the Mn­(L)–Mn1­(L) distance across the edge of two edge-sharing MnO_6_ octahedra (Mn­(L)­O_4_–O_2_–Mn1­(L)­O_4_ linkage). The third peak at ca. 3.43 Å is associated with the Mn­(L)–O_2_ distance and the Mn­(L)–Mn1­(IL) distance across the corner of two corner-sharing Mn­(L)­O_6_ and Mn­(IL)­O_6_ octahedra at a vacancy site (Mn­(L)­O_5_–O–Mn1­(IL)­O_5_ linkage, Figure 8). The Mn­(IL)­O_6_ octahedra share three corners with Mn­(L)­O_6_ octahedra (triple-corner-sharing, TCS, interlayer position). There is no correlation at 4.1–4.2 Å, suggesting the absence of Mn1­(IL)–Mn1­(IL) TCS pairs across vacancies. Consequently, the vacancies are primarily singly capped. Peaks at longer distances correspond to higher-order Mn–O and Mn–Mn correlations.

**Figure 8:**
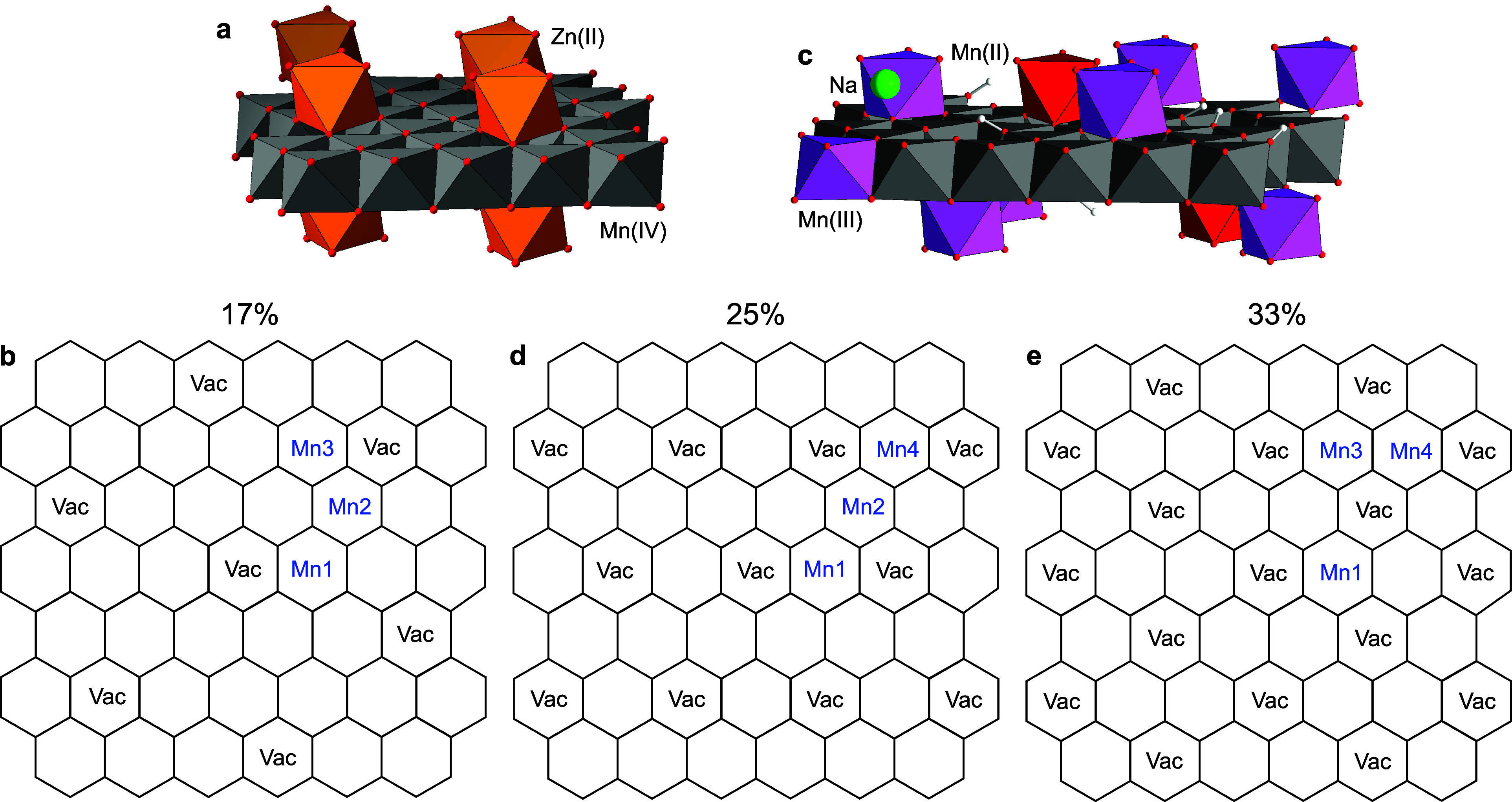
Polyhedral structures and schematic representation of the ordered distribution of vacancies in a phyllomanganate layer. (a,b) Chalcophanite (ZnMn_3_O_7_·3H_2_O) structure. 17% of the Mn sites are vacant (every 4th Mn shell). (c,d) Model structure of δ-MnO_2_^HE^ at low IS. 25% of the Mn sites are ideally vacant (every 3rd Mn shell). (e) Maximum density of vacancy sites (33%, every 2nd Mn shell is vacant).

The PDFs of δ-MnO_2_-6 and δ-MnO_2_-8, as well as the PDFs of low-IS δ-MnO_2_^HE^-6 and δ-MnO_2_^HE^-8, mainly have similar shapes, with only slight amplitude differences (Figure 7a,b). According to XRD, δ-MnO_2_-8 contains more Mn­(IL) than δ-MnO_2_-6 (Table 1). This difference appears in the PDF as higher peaks at 3.43 Å (Mn­(L)–Mn1­(IL)) and 5.30 Å (Mn­(L)–Mn2­(IL)) for δ-MnO_2_-8 (Figure 7a). Notably, there is an inverse trend in the amplitudes of the Mn­(L)-Mn1,2 (L) peaks at ca. 2.86 and 4.95 Å, on one side, and the amplitudes of the Mn­(L)–Mn1,2­(IL) peaks at ca. 3.43 and 5.30 Å, on the other side, between δ-MnO_2_ and δ-MnO_2_^HE^ (Figure 7c,d). This clearly shows that low-IS δ-MnO_2_^HE^ has less Mn­(L) and more Mn­(IL) than δ-MnO_2_, consistent with the XRD simulation (Table 1).

Using also PDF, we showed in 2013 that the Mn­(L)-Mn­(L) correlations decrease, and the Mn­(L)–Mn­(IL) correlations increase, when δ-MnO_2_ is equilibrated at low pH (Figure 7e).^58^ At pH 3, δ-MnO_2_ (δ-MnO_2_-3) contained 21% Mn­(IL). Figure 7f shows that the δ-MnO_2_-3 and low-IS δ-MnO_2_^HE^-6/8 PDFs are similar, aligning well with the 23% Mn­(IL) calculated here by XRD for δ-MnO_2_^HE^ (Table 1). Therefore, HEPES acts both as a source of electrons^54^ and protons.^118^ It releases at circumneutral pH as much Mn­(L) into the interlayer as protons at pH 3, but through a different mechanism. Interlayer Mn­(III) cations result from the reduction of lattice Mn­(IV) cations with HEPES. Conversely, at low pH, they form through disproportionation.^99,109,119^ Ling et al.^118^ demonstrated that HEPES can serve as a proton source to convert TcBi to HBi at circumneutral pH. Reduction of layer Mn­(IV) and migration of Mn­(III) out of the δ-MnO_2_ layer can also be achieved with the MES buffer (2-(*N*-morpholino)­ethanesulfonic acid, C_6_H_13_NO_4_S). Still, the reduction is lower with MES than with HEPES.^78^

#### 2.1.4 Structural Model

Based on XRD fitting results, one out of four Mn­(L) octahedral sites is empty (25%) in the low-IS δ-MnO_2_^HE^-6/8 layers (Table 1). Because two nearest vacancy sites would cause unfavorable steric strain within a MnO_2_ layer, leading to local structural disruption, assuming some semiordering of the Mn vacancies seems appropriate. A comparison can be made with the phyllomanganate chalcophanite (ZnMn_3_O_7_·3H_2_O), in which one out of seven octahedral sites is vacant (Figure 8a).^120^ The 4+ charge deficit from a missing Mn­(IV) cation is compensated by two interlayer Zn­(II) ions on each vacancy side. According to PDF results, vacancies are singly capped by Mn­(II)/Mn­(III) cations in low-IS δ-MnO_2_^HE^6/8, so the 4+ charge deficit should be balanced by the coadsorption of monovalent cations, such as Na­(I) and H^+^, similar to HBi^110^ (Table 1). In chalcophanite, every fourth Mn atomic shell is empty, resulting in a vacancy density of 14% (Figure 8a). Figure 8b shows that the desired density of approximately 25% in low-IS δ-MnO_2_^HE^-6/8 layers is obtained by removing the Mn atoms from every third Mn atomic shell. Consequently, we propose the model shown in Figure 8b for the ideal composition and structure of low-IS δ-MnO_2_^HE^-8 (Table 1).

Building on this reasoning, Figure 8c shows that the maximum density of vacancies, and thus of Mn­(IL), cannot exceed 33% and is reached when the second Mn shell is empty. Interestingly, the Mn­(III) cations in the phyllomanganate lithiophorite ((Al_0.67_Li_0.32_)­(Mn_0.68_^4+^Mn_0.32_^3+^)­O_2_(OH)_2_) exhibit the same distribution pattern as the 33% vacancy model (Figure 11 in ref 17). Examples like chalcophanite and lithiophorite, as well as TcBi and HBi,^99,121^ demonstrate that cation and vacancy ordering is common in phyllomanganates. Moreover, Drits et al.^121^ showed in 2002, using SAED, that the distribution of the vacant sites in HBi can be rearranged in various ways by capping them with Cu, Zn, or Pb in metal sorption experiments.

Recognizing the complexity of δ-MnO_2_ and the inevitable oversimplification of the resulting models, the structural flexibility of phyllomanganates supports a certain degree of ordering of Mn­(IL) and vacant sites in low-IS δ-MnO_2_^HE^. Under transmission electron microscopy (TEM) observation, low-IS δ-MnO_2_^HE^-8 appears as an aggregation of thin flakes 20–70 nm wide, with some long, flexible, rolled particles measuring 200–500 nm in length (Figures 9 and S4). The flakes and rolled particles include 3–6 layers, and the flakes contain randomly oriented nanocrystalline domains of 3–6 nm. The size of the nanocrystals is close to the diameter of the coherent scattering domains (CSD) determined by XRD (6.5 nm, Table S3), indicating that they are monodomain. The larger crystallites produce spotty reflections in SAED over powder-like continuous rings from smaller crystallites. Overall, little structural difference is observed compared to the nontreated δ-MnO_2_ (Figures 1 and S1), except for the rolled particles.

**Figure 9:**
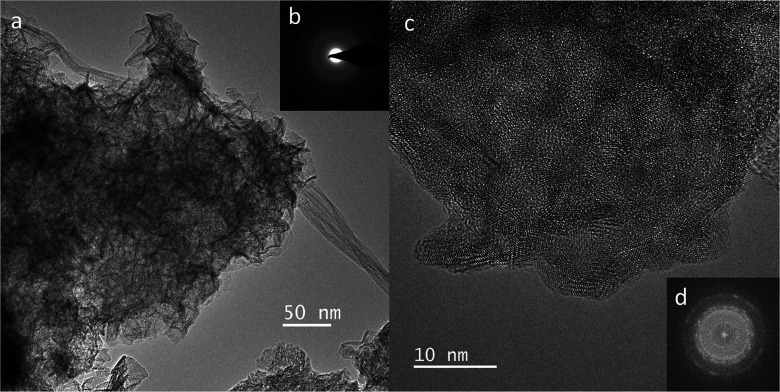
Transmission electron images and diffraction of low-IS δ-MnO_2_^HE^-8. (a) Low-resolution image of an aggregate. (b) Corresponding SAED pattern showing only diffused rings from extremely low crystalline materials. (c) HRTEM image of a flake composed of nanograins randomly oriented in the *ab*-plane. (d) Fast-Fourier Transform (FFT) of the platelet image, confirming the occurrence of nanocrystallized domains within an amorphous matrix.

### 2.2 Structure of δ-MnO_2_^HE^ at High Ionic Strength

#### 2.2.1 Chemical Composition

The chemical compositions of δ-MnO_2_-6/8 and δ-MnO_2_^HE^-6/8 synthesized at 0.6 M NaCl ionic strength (IS) are reported in Table 2. The Na/Mn atomic ratios are high, ranging from 0.42 to 0.61. We show below that some sodium is precipitated as NaCl, which prevents the calculation of chemical and structural formulas. Excess Na did not impact the reducing ability of HEPES. High-IS δ-MnO_2_-6/8 have AMOS values of 3.89–3.92, while high IS δ-MnO_2_^HE^-6/8 have AMOS values of 3.59 (pH 6) and 3.71 (pH 8) (Table 2). The AMOS values and the fractions of Mn­(III) and Mn­(II) are close to those observed at low IS (Tables 1 and S1). We show below that, despite these similarities, δ-MnO_2_^HE^ has distinct structures at low and high IS.

**Table 2:** Chemical Composition and AMOS of δ-MnO_2_-6/8 and *δ*-MnO_2_^HE^-6/8 at 0.6 M NaCl

**sample**	**chemical composition** ^a^	**AMOS** ^b^
δ-MnO_2_-6 – high IS	Na_0.42_^+^Mn_0.01_^2+^Mn_0.09_^3+^Mn_0.90_^4+^O_2_ · 0.83H_2_O	3.89
δ-MnO_2_-8 – high IS	Na_0.45_^+^Mn_0.01_^2+^Mn_0.07_^3+^Mn_0.92_^4+^O_2_ · 0.75H_2_O	3.92
δ-MnO_2_^HE^-6 – high IS	Na_0.61_^+^Mn_0.06_^2+^Mn_0.29_^3+^Mn_0.65_^4+^O_2_ · 0.55H_2_O	3.59
δ-MnO_2_^HE^-8 – high IS	Na_0.57_^+^Mn_0.03_^2+^Mn_0.22_^3+^Mn_0.74_O_2_ · 0.47H_2_O	3.71

a The high Na content is due to the precipitation of NaCl.

b From chemical analysis (Table S1).

#### 2.2.2 High-Energy XRD

The XRD pattern of high-IS δ-MnO_2_^HE^-8 features sharp NaCl lines, two strong 00*l* reflections, and weak *hkl* reflections to the left and right sides of the 20,11 *hk* reflection and the left of the 02,31 *hk* reflection (Figure 5b). The 00*l* reflections are less intense at pH 6 than pH 8, and the *hkl* reflections appear as small modulations. The *d*(*hkl*) spacings of the well-defined *hkl* reflections are 2.15, 1.82, 1.64, 1.54, 1.47, 1.44, and 1.42 Å, matching those of TcBi.^101^ Because TcBi has an AMOS of 3.69, while the initial δ-MnO_2_-6/8 have an AMOS of 3.89–3.92 when equilibrated at 0.6 M NaCl IS (Table S1), the formation of TcBi crystallites involves the partial reduction of lattice Mn­(IV) to Mn­(III) by HEPES and an increase in stacking order along the *c*-axis. The rotational stacking order of the diffracting crystallites is aided by weakly bonded interfacial Na­(I) ions acting as charge-balancing cations.^98^ The 02,31 reflection now appears asymmetric, indicating a change in layer symmetry from hexagonal to orthogonal. The Mn­(III) and Mn­(IV) cations are distributed in the layers with some degree of TcBi-type ordering (Figure 3b).

These results align with earlier observations by Elzinga and Kustka,^98^ who found that HEPES reduces Mn­(IV) to Mn­(III) and decreases the layer symmetry. They did not observe the appearance of *hkl* reflections, likely because their experiment lasted 2 days and was carried out at a lower Na concentration (0.1 M). However, the experiment was performed in an anaerobic glovebox, and O_2_ depletion accelerates the δ-MnO_2_ to TcBi transformation, based on our own experience. We surmise that the lower Na concentration was the limiting factor in the formation of large TcBi crystals in the Elzinga and Kustka^98^ experiment.

#### 2.2.3 PDF

The main differences between the PDFs of δ-MnO_2_ and δ-MnO_2_^HE^ at low and high IS are the presence of pairwise correlations from NaCl and the absence of a Mn2­(IL) peak at 0.6 M IS, confirming the lack of Mn­(IL) at high Na concentration (Figure 10). The Na–Na, Na–Cl, and Cl–Cl pairs either overlap with the Mn–Mn, Mn–O, and O–O pairs or are weak, except at 4.0 Å, where the first Na–Na and Cl–Cl pairs are observed distinctly.

**Figure 10:**
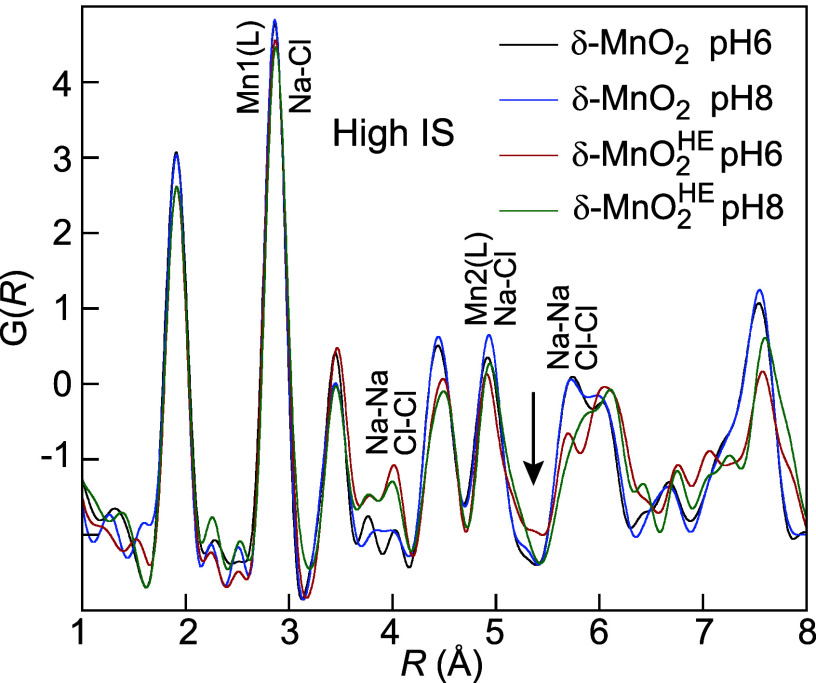
PDF of δ-MnO_2_-6/8 and δ-MnO_2_^HE^-6/8 at high IS. The arrow indicates the position of the Mn2­(IL) peak at low IS (Figure 7).

#### 2.2.4 EXAFS Spectroscopy

One intriguing question is whether the orthogonal symmetry of the δ-MnO_2_^HE^ layers observed by XRD at high IS causes a wave beating at *k* = 7.9 Å^–1^ in the EXAFS spectra, as seen for TcBi (Figure 4a). The “dunce’s cap” shape of the photoelectron wave at this *k* value was not observed by Simanova et al.^102^ after 2 days of reaction at low IS. This question was further explored by measuring the EXAFS spectra of δ-MnO_2_-6/8 aged for 3 days and high-IS δ-MnO_2_^HE^-6/8 aged for 7 days at high IS. All four EXAFS spectra exhibit a similar shape, matching all features of HBi (Figure 11a). No double antinode is observed at *k* = 7.9 Å^–1^, even for high-IS δ-MnO_2_^HE^-8, which shows a clear departure from hexagonal layer symmetry by XRD. The only notable difference among the δ-MnO_2_-6/8 and high-IS δ-MnO_2_^HE^-6/8 spectra is a reduction in the EXAFS amplitude for δ-MnO_2_^HE^. This observation may be explained by a greater disorder in the Mn–O and Mn–Mn distances due to the higher content of layer Mn­(II,III) cations in high-IS δ-MnO_2_^HE^ (∼25–35%, Table 2). The Mn­(III) cations are not well ordered in rows as in TcBi (Figure 3b); otherwise, a double antinode at *k* = 7.9 Å^–1^ would be observed (Figure 4a). However, the intense 00*l* lines in XRD indicate that the layer stacking is long-range ordered along the *c* direction. At the same time, the discernible *hkl* reflections suggest some semiordering of the Na­(I) cations in the interlayer space and of the Mn­(III) and Mn­(IV) cations within the layer. Due to the Jahn–Teller distortion of the Mn­(III)­O_6_ octahedra, a random distribution of ∼25–35% Mn­(II,III) would lead to unfavorable steric strain within the MnO_2_ layer.^99^ This raises the question of how sensitive EXAFS is to the Mn­(III) distribution pattern. To explore this, we first need to review which Mn–Mn atomic pairs cause the wave beating in TcBi.^17^

**Figure 11:**
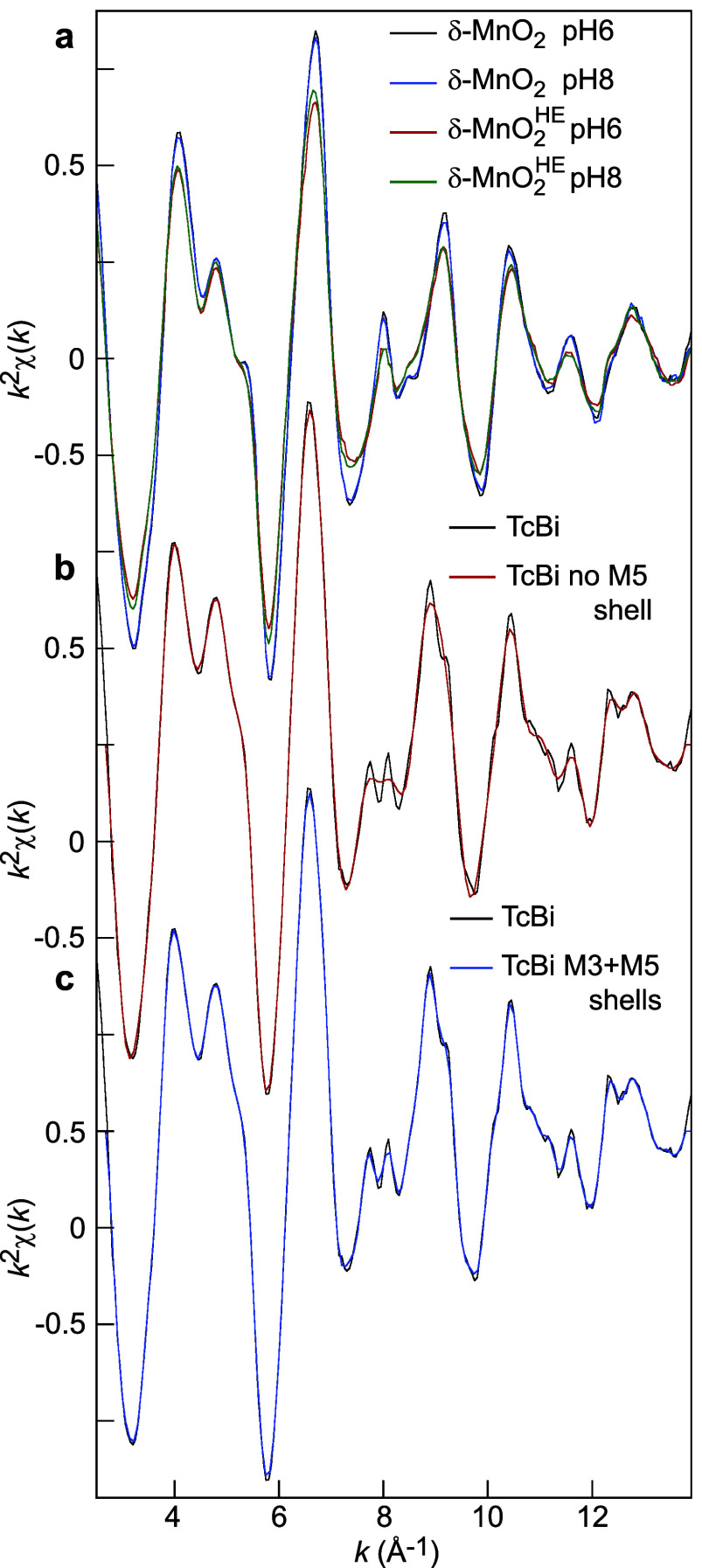
Mn K-edge EXAFS spectra. (a) δ-MnO_2_-6/8 and δ-MnO_2_^HE^-6/8 at high IS. (b) Experimental TcBi spectrum (black) and Fourier-filtered spectrum without the Mn5 shell (red). (c) Experimental TcBi spectrum (black) and Fourier-filtered spectrum with the Mn3 and Mn5 shells (blue).

In structures with three or more atoms in a collinear arrangement, forward scattering through the center atom(s) enhances the backscattering from the distant atoms. This so-called “focusing effect” is sensitive to any angular deviation from perfect alignment and to changes in interatomic distances. In phyllomanganates, the Mn atoms in the Mn3 shell are aligned along the axis of three edge-shared octahedra (*d*(Mn–Mn3) = 2 × *d*(Mn–Mn1) ≈ 5.70 Å). The Mn atoms in the Mn5 shell are aligned along the axis of four edge-sharing octahedra ((*d*(Mn–Mn5) = 3 × *d*(Mn–Mn1) ≈ 8.55 Å, Figure 12a). The population histogram of the Mn–Mn distances shows that all distances within a shell are equal when the layer has hexagonal symmetry (e.g., HBi), and differ when the symmetry is orthogonal: Δ*R*(Mn3) = 0.20 Å and Δ*R*(Mn5) = 0.30 Å for TcBi (Figure 12b,c).^101^ As a result, the Mn3 and Mn5 peaks form a doublet in the radial distribution function of TcBi (Figures 4b and 13). In *k*-space, the split distances of the Mn3 shell create an antinode of the EXAFS photoelectron wave at *k* = π/(2Δ*R*) ≈ 7.9 Å^–1^ (Figures 4a and S5a). Data analysis indicates that the double antinode pattern is due to the Mn5 electronic wave, which has a maximum amplitude at 7.8 and 8.1 Å^–1^ and a minimum (i.e., negative amplitude) at 7.95 Å^–1^. When the M5 shell is removed by Fourier backtransformation, there is a single antinode (Figure 11b), and including the Mn3 and Mn5 shells in the backtransform restores it (Figure 11c). From these observations, we conclude that the shape of the EXAFS signal at 7.9 Å^–1^ can serve as a fingerprint of the average distribution of the Mn­(III) and Mn­(IV) cations within the MnO_2_ layer. Our findings align with Ling et al.’s^122^ results obtained on Ca-birnessite through X-ray diffraction and EXAFS spectroscopy.

**Figure 12:**
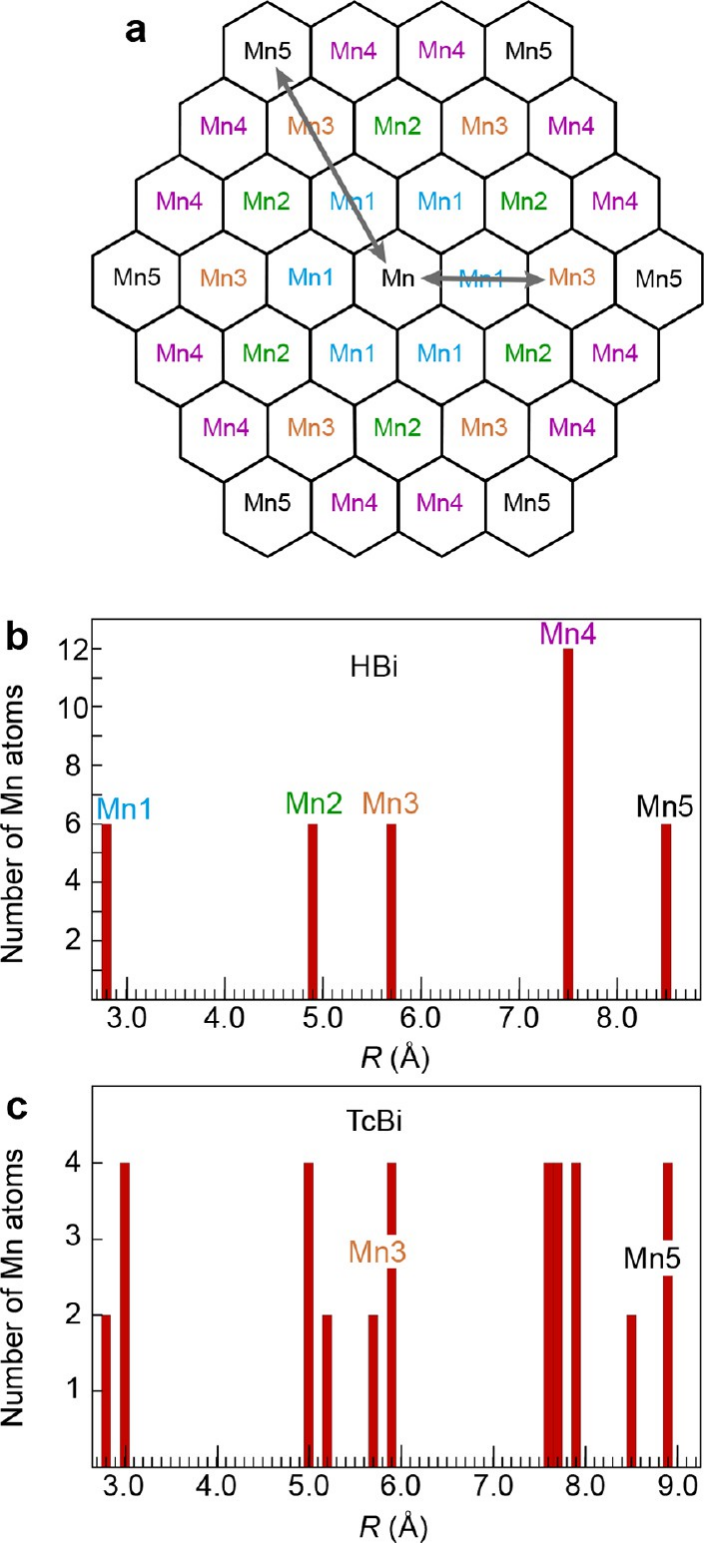
(a) Mn–Mn atomic shells in a MnO_2_ layer. Arrows indicate the Mn–Mn3 and Mn–Mn5 multiple-scattering paths. Population histograms of the Mn–Mn distances in hexagonal birnessite (b),^119^ and triclinic birnessite (c).^101^

**Figure 13:**
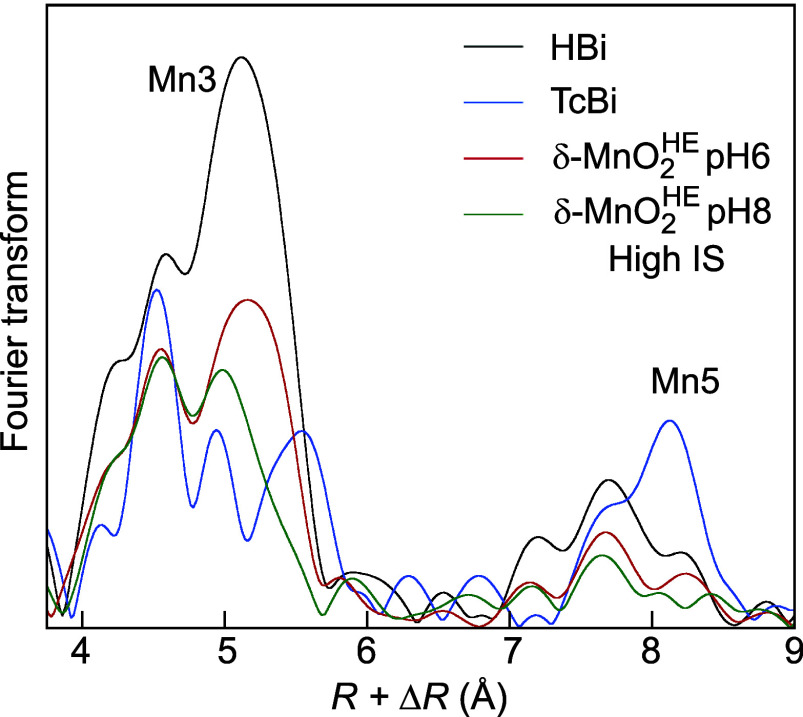
Fourier transforms of HBi, TcBi, and δ-MnO_2_^HE^-6/8 at the scattering distance of the Mn3 and Mn5 shells.

Using the Mn3 and Mn5 shells as indicators for the ordering of Mn­(IV) and Mn­(III), Figure 13 shows that the amplitude of the Mn3 and Mn5 peaks for high-IS δ-MnO_2_^HE^-6 and δ-MnO_2_^HE^-8 falls between those of HBi and TcBi. The shoulder on the right side of the Mn3 peak for δ-MnO_2_^HE^-8 is suggestive of split Mn–Mn3 distances. A good fit of the EXAFS contribution from the Mn3 shells was achieved with a single Mn shell at 5.65 Å for δ-MnO_2_^HE^-6 and with two Mn subshells at 5.60 and 5.73 Å for δ-MnO_2_^HE^-8 (Δ*R*(Mn3) = 0.13 Å, Figure S5b,c). The 0.13 Å split distance exceeds the experimental resolution of Δ*d* = π/2*k*_max_ = π/(2 × 14) = 0.11 Å.^123^ The antinode of δ-MnO_2_^HE^-8 is at *k* = π/(2 × 0.13) = 12.0 Å^–1^, which explains its absence at the *k* = π/(2Δ*R*) ≈ 7.9 Å^–1^ indicator region (Figure 11a). Therefore, the Mn3 shell is a sensitive spectroscopic probe of the decreasing layer symmetry from hexagonal to orthogonal, as observed by XRD (Figure 5b). It is responsive to the split of the Mn–Mn distances caused by a cooperative Jahn–Teller effect, in which the Mn­(III) octahedra are ordered at short distances in the MnO_2_ layer with their elongated bonds aligned in the same direction (Figure 3b). We conclude that an onset of short-range Mn­(IV)–Mn­(II/III) ordering is detected by EXAFS at pH 8, whereas there is no evidence of this at pH 6. However, circumstantial evidence suggests some long-distance heterogeneous distribution of Mn­(III) and Mn­(IV) at pH 6, based on observations of weak *hkl* reflections (Figure 5b). High-IS δ-MnO_2_^HE^-8 was examined using HRTEM and SAED to further explore its nanostructure.

#### 2.2.5 HRTEM and SAED of δ-MnO_2_^HE^-8

High-IS δ-MnO_2_^HE^-8 comprises nanoflake aggregates having low Na content (Na/Mn atomic ratio = 0.07–0.10) and large platelet- and lath-shaped crystals, which are several hundred nm in size and have high Na content (Na/Mn 0.20–0.28, Figures 14, 15a, and S6–14). The aggregates resemble those in untreated δ-MnO_2_ (Figure 1) and low-IS δ-MnO_2_^HE^ (Figure 9). The flakes remained 30–70 nm wide but are thicker, composed of 6–10 layers, and the randomly oriented nanocrystals in the basal planes now measure 5–10 nm, compared to 3–4 nm for δ-MnO_2_ and 3–6 nm for low-IS δ-MnO_2_^HE^. The large plates contain between 10 and 20 layers (Figures S7 and S8). The increase in flakes’ thickness in both types of crystals explains the enhanced 001 reflection in XRD (Figure 5b). Two types of SAED patterns were observed in the flakes aggregates: some display only continuous rings (Figures 14b and S6c), while others also show spotty reflections from the *hkl* reflections of TcBi (Figures 14f, 15b–h, S11, S12, and S14).

**Figure 14:**
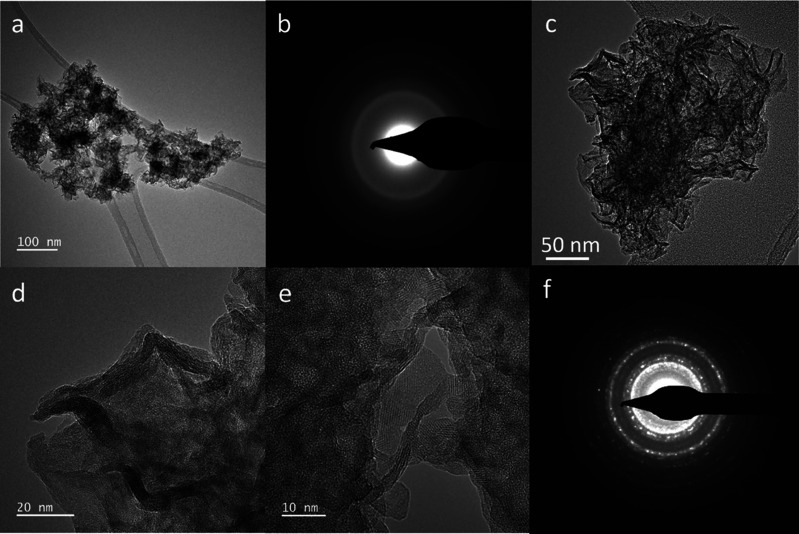
Transmission electron images and diffraction of high-IS δ-MnO_2_^HE^-8. (a,b) Image and SAED pattern of a flakes aggregate showing only diffuse rings from low crystalline nanoparticles. (c–e) Low- and high-resolution images of a better crystallized polycrystalline aggregate. The flakes contain about 6–10 layers and are composed of nanograins randomly oriented in the *ab*-plane. (f) SAED pattern from (c) showing the presence of *hkl* reflections from TcBi crystallites.

**Figure 15:**
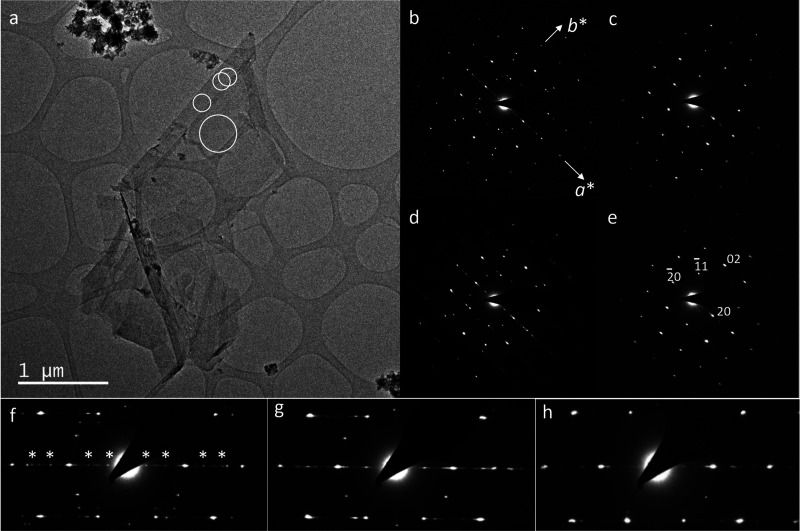
Transmission electron images and diffraction of high-IS δ-MnO_2_^HE^-8. (a) Image of a flakes aggregate and large crystals. (b) SAED pattern of a large single crystal showing a pseudo hexagonal symmetry of the *ab* plane, along with satellite reflections caused by partially ordered interlayer cations. (c–e) SAED patterns of small areas circled in (a) located at the edge of the large crystal, illustrating the local variation of superstructure. (f–h) Enlarged views of the central part of the SAED patterns in (b–d), respectively, with the *A* = 3*a* superlattice reflections (star symbols in (f)) replaced with incommensurate reflections in (g).

The 5–10 nm nanocrystals are also found within some larger crystals, usually near the edges or in less ordered regions (Figures 16a, S9, S10, and S12). Their lattice fringes mostly align parallel to the *b* direction, although some wavy alignments are observed. The nanostructural features suggest that crystal growth occurred via crystallographically oriented attachment of nanocrystals with distinct cation distributions (Figures S9, S10, and S12). There is a high degree of structural heterogeneity, with domains of semioriented nanocrystals, at one end (Figures 16a, S9, and S10), and extended regions of parallel lattice fringes, at the other (Figures 16b,c, S14, and S15).

**Figure 16:**
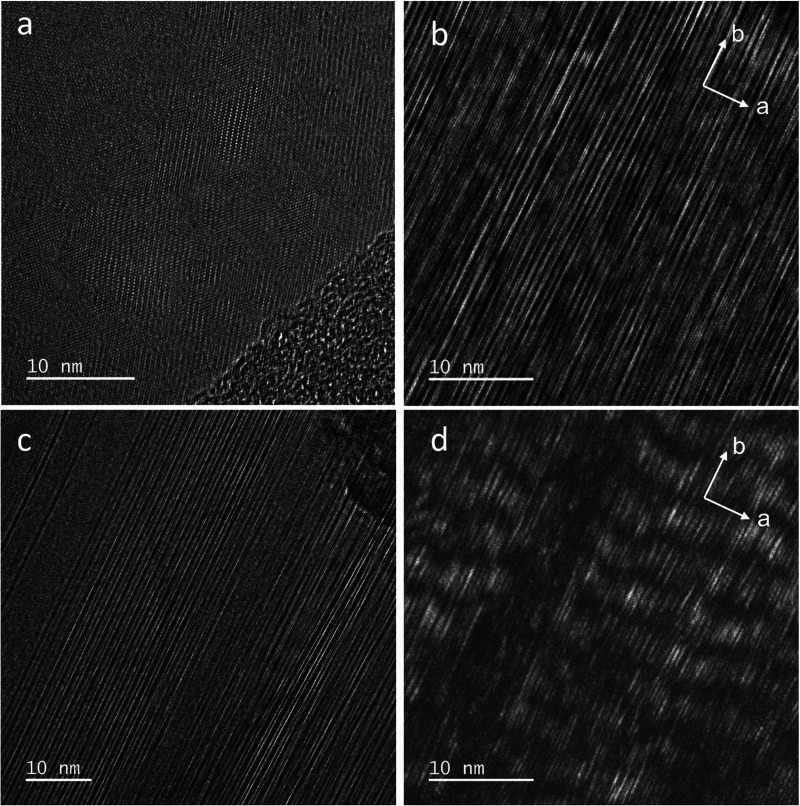
High-resolution transmission electron images of the basal planes of high-IS δ-MnO_2_^HE^-8 crystals. (a) Semioriented nanocrystals. (b) Aligned lattice fringes in the *a* direction resulting from the oriented growth of nanocrystals. The nanostructure is discontinuous in the *b* direction. (c) Variation in the size of the nanodomains in the *a* direction, explaining the streaks observed in SAED. (d) Dark-field image of (b), showing the zebra-shaped periodicity of the coherent scattering domains along *b*. The length of the zebra stripes along *a* matches the width of the lattice fringes in (b).

In well-ordered regions, regular intensity contrast is observed along the *a* direction, revealing cation ordering (Figure S14e). The width of the stripes, where the superlattice fringes remain evenly spaced, varies greatly (Figures 16c, S14e, and S15). The lattice fringes are interrupted or weakened after some distance along the *b* direction. In some crystals, they undulate with a periodicity of about 5–7 nm (Figure S15), which aligns with the diameter of the coherent scattering domains (CSD) for low-IS δ-MnO_2_^HE^ obtained by XRD (Table S3), and an angle of ∼3–8° (Figures S10 and S13–S15). This is likely caused by nonrelaxed lattice distortions resulting from partially ordered Mn­(III) distribution. The fringe undulations along *b* observed in HRTEM imaging produce a characteristic zebra-shaped contrast pattern with a 5–7 nm periodicity in dark-field imaging (Figures 16d and S15). The length of the zebra stripes in the *a* direction matches the width of the fringe stripes observed by HRTEM imaging.

The nanostructural heterogeneity causes notable variations in nanodiffraction patterns both within and between individual crystals (Figures 15b–h, S6d–f, S11b, S12b, and S14b). Most SAED patterns from individual crystals display diffuse streaks and elongated spots arranged in arrays that are consistent with highly disordered, close-packed anionic sheets in the [100]* direction, along with an irregular distribution of Na and Mn­(III)/Mn­(IV). Super-reflections with A* = a*/3 and A* = a*/6 were observed in some areas of the high-IS δ-MnO_2_^HE^-8 crystals (Figures 15f,h, S14c, and S16a), indicating some TcBi-type ordering of the cooperative Jahn–Teller distortion, which was also identified by XRD and EXAFS (Figure 3b).

Electron diffraction is sensitive to the superlattice arrangement of the Na cations in the MnO_2_ interlayer. Still, their distribution mainly reflects the Mn­(IV)/Mn­(III) ordering within the layer, due to local charge balance.^101^ This ordering is responsible for the HRTEM superlattice fringes in the *a* direction, as confirmed by electron diffraction mapping using 4D-STEM and virtual dark-field images reconstructed with different super-reflections (Figure S16). No streaking or super-reflection has been observed in the *b* direction, as the lattice fringes are interrupted after a certain length along *b*, except for satellites in a few cases (Figures 15f,g and S6d). The super-reflections and satellites in the SAED patterns are not multiple-scattering effects, because these effects are observed only in highly ordered, thick samples containing heavy elements.^124^ In addition, double diffraction, if it occurs, can add intensity to an *hkl* reflection that would otherwise be extinct by symmetry. It cannot generate additional reflections (i.e., super-reflections) between the main-cell lattice reflections, unlike superlattice reflections from superstructures.

## 3 Conclusion

A variety of methods, including chemical analysis, high-energy X-ray diffraction measurement and modeling, PDF, EXAFS spectroscopy, EXAFS spectroscopy, HRTEM, and SAED have been used to analyze the structure of δ-MnO_2_ reacted with HEPES at pH 6 and 8, under both low (<0.01 M NaCl) and high (0.6 M NaCl) ionic strength conditions. The findings reveal an unexpected perspective on reaction products. At low IS, HEPES functions both as an electron donor, reducing Mn­(IV) to Mn­(III) and Mn­(II), and as a proton donor, resulting in a structure at pH 8 similar to hexagonal δ-MnO_2_ equilibrated at about pH 3 without the presence of organic reductants. The low-IS δ-MnO_2_^HE^ layer has approximately 23% vacancies at pH 6 and 8, all capped with major Mn­(III) (17–19%) and minor Mn­(II) (4–6%) cations in the interlayer.

The presence of interlayer Mn­(III) affects the reactivity of δ-MnO_2_ in two opposite ways. On one side, Mn­(III) blocks access to vacancy sites for other cations, decreasing its sorption capacity. In the case of thallium, Mn­(III) prevents Tl­(I) from sorbing on vacancies and reduces its oxidation to Tl­(III) by Mn­(IV).^31,62,108^ On the other side, Mn­(III) can boost redox reactions involving single electron transfer. For example, it can facilitate the oxidation of Co­(II) to Co­(III), while interlayer Mn­(III) is reduced to Mn­(II).^107,125^ Therefore, understanding the structure of δ-MnO_2_^HE^ at low IS should help advance studies on the sorption and redox reactions of metals that are sensitive to the amounts of Mn­(III) and vacancies.

When δ-MnO_2_ reacts with HEPES at 0.6 M NaCl IS, no proton is adsorbed, and the hexagonal symmetry shifts to the triclinic symmetry characteristic of TcBi,^100,102^ which is typically obtained by controlled oxidation of highly alkaline Mn­(II) hydroxide suspensions.^99,101,126^ High-IS δ-MnO_2_^HE^-6/8 contains no interlayer Mn, and its layer has 22–29% Mn­(III) and 3–6% Mn­(II). Na­(I) in the interlayer fully compensates for the layer charge deficiency. The transition from hexagonal to triclinic structure is more prominent at pH 8 than at pH 6, consistent with the synthesis of TcBi in an alkaline medium. The change in layer stacking from turbostratic (δ-MnO_2_) to triclinic (high-IS δ-MnO_2_^HE^) indicates an ordering of the Na­(I) and Mn­(II/III) cations in the transformed diffracting crystals. However, not all δ-MnO_2_ crystals rearrange simultaneously, and high-IS δ-MnO_2_^HE^ is a mixture of ordered, semiordered, and disordered δ-MnO_2_ nanocrystals. This heterogeneity is not captured by bulk-averaging PDF and EXAFS methods, but is observed by HRTEM coupled to nanodiffraction. The EXAFS signal is a one-dimensional projection in reciprocal space of a bulk-averaged three-dimensional structure. The onset of in-layer Mn­(IV)/Mn­(III) ordering is compressed in EXAFS data and is not easily accessible. Analysis of the Mn3 shell can provide a hint of symmetry lowering before the double antinode fingerprint appears. The lower sensitivity of EXAFS compared to XRD to departure from hexagonal layer symmetry explains why the nascent in-layer ordering was not observed by Simanova et al.^102^ Further research is needed to examine the effects of Na concentration, aging, and temperature on the kinetics of the in-layer ordering and complete transformation of δ-MnO_2_ to TcBi.

Well-crystallized TcBi can be synthesized with other interlayer cations besides Na, including K, Cs, Mg, Ca, and Sr.^97,104,127^ Our results suggest that triclinic δ-MnO_2_^HE^ can also be obtained by the incorporation of these alkali and alkaline earth metal ions in the interlayer at high IS. Here, the hexagonal δ-MnO_2_ to triclinic δ-MnO_2_^HE^ phase transformation was induced by the HEPES-triggered reduction of Mn­(IV) to Mn­(III). Migration of Mn­(III) into the interlayer, and of Mn­(II) through the disproportionation of Mn­(III) into Mn­(II) and Mn­(IV), which occur at low IS, are impeded by Na­(I) at high IS. Previous studies showed that a similar phase transformation can be achieved through the production of Mn­(III), not from HEPES, but from the comproportionation of Mn­(II) sorbed on vacancies and layer Mn­(IV), followed by the migration of Mn­(III) into the vacancies and its subsequent ordering in the MnO_2_ layer.^78,128,129^ Lastly, the disorder-to-order structural transformation described here can also be promoted by adding pyrophosphate to form Mn­(III)-PP complex during the photochemically assisted formation of δ-MnO_2_ nanosheets.^115^

## 4 Materials and Methods

### 4.1 Sample Preparation and Chemical Characterization

#### 4.1.1 Synthesis of δ-MnO_2_ and δ-MnO_2_^HE^

All chemicals were of analytical grade or higher and purchased from Sigma-Aldrich. The synthesis of δ-MnO_2_ and δ-MnO_2_^HE^ followed established protocols.^87,102^ 250 mL of 0.15 M MnCl_2_ were pumped with a peristaltic pump into 250 mL of 0.1 M KMnO_4_ and 0.2 M NaOH at a rate of 20 mL min^–1^. The mixture was continuously stirred in the dark at room temperature for at least 24 h. The suspension was split into 50 mL centrifuge tubes, centrifuged at 5000 rpm for 5 min, rinsed 3 times with 1 M NaCl to replace K^+^, and rinsed 3 times with ultrapure water. Then, the solid was lyophilized for 2 d until reaching a constant weight. For δ-MnO_2_^HE^ synthesis, a suspension of 0.72 g L^–1^ δ-MnO_2_ was added to a solution of 0.01 M HEPES and 0.01 M NaCl at pH 7.0 (v/v = 1:1). The final total Mn concentration in the mixed suspension was approximately 0.04 M. The mixture was continuously stirred in the dark under ambient atmosphere for 48 h. The solid was rinsed and lyophilized as with δ-MnO_2_.

#### 4.1.2 Equilibration of δ-MnO_2_ and δ-MnO_2_^HE^

To examine the effects of salinity and pH on the structure of δ-MnO_2_ and δ-MnO_2_^HE^, suspensions (1 g L^–1^) of the two MnO_2_ samples were equilibrated at pH 6 and 8 under buffered conditions that simulate lacustrine (<0.01 M NaCl, low IS) and seawater (0.6 M NaCl, high IS) environments for 3 d (δ-MnO_2_) or 7 d (δ-MnO_2_^HE^). For the low IS system, the MnO_2_ solids were suspended in ultrapure water, and the pH was adjusted with 0.01 M HCl (pH 6) and NaOH (pH 8), using a potentiometric titrator (905 Titrando, Metrohm). For the high IS system, the solids were added to a 0.6 M NaCl solution, and the pH was adjusted with 0.01 M NaOH (pH 6 and 8). During equilibration, less than 5 mL of HCl (low IS, pH 6) and approximately 50 mL of NaOH (all other conditions) were added. The resulting solids were lyophilized as previously described. Samples were stored in tubes covered with Al foil and kept at −80 °C.

#### 4.1.3 Chemical Analyses

The δ-MnO_2_ and δ-MnO_2_^HE^ samples (0.01 g) were digested in 20 mL of 0.25 M NH_3_OH.Cl, and their Na and Mn contents were measured using inductively coupled plasma atomic emission spectroscopy (ICP-AES 720ES, Varian, ISTerre, University of Grenoble-Alpes, and iCap 6500, Thermo Fisher Scientific, Mikroanalytisches Labor Pascher) and visible spectrophotometry (Method 8149, DR1900, HACH), respectively. Strongly adsorbed (structural) water content was determined by thermogravimetric analysis (TGA) based on weight loss of approximately 20 mg powder. Thermal analysis was performed with a thermal microanalyzer (Mettler Toledo, ISTerre, University of Grenoble-Alpes) at a heating rate of 10 °C min^–1^ up to 400 °C under an N_2_ flow rate of 20 mL min. Weight losses were calculated within the 80–250 °C temperature range. Differential scanning calorimetry (DSC) analysis showed that δ-MnO_2_ and TcBi exhibit an exothermic peak at 80 °C attributed to surface-adsorbed species.^101,110^

The average Mn oxidation states (AMOS) of the eight equilibrated MnO_2_ samples were determined using the pyrophosphate (PP) extraction method.^130^ Mn­(III) was selectively extracted with PP at pH 6.7 for 24 h at a solid-to-solution ratio of 1:25. The suspension was filtered, and the PP-complexed Mn­(III) was measured by UV–vis spectrophotometry at 254 nm. The total dissolved Mn content was assessed using visible spectrometry (Method 8149, DR1900, HACH), while Mn­(II) was calculated from a mass balance. The standard deviations (SD) of Mn­(II) and Mn­(III) in Table S1 were obtained from triplicate measurements.

### 4.2 High-Energy X-ray Diffraction

XRD data were collected at the European Synchrotron Radiation Facility (ESRF) on beamline ID22.^131^ Measurements were performed using borosilicate glass capillaries in Debye–Scherrer geometry, with a Si 111 monochromated beam and a PerkinElmer XRD1611 detector. Scattering data were collected in transmission mode at 35 keV (0.35–5.3 Å^–1^ Q-range) and 70 keV (0.5–25 Å^–1^ Q-range). The detector geometry was calibrated with the LaB_6_ standard using pyFAI 2D.^132^ Data measured at 35 keV were modeled with CALCIPOW,^133–135^ as described previously,^58^ and data measured at 70 keV were used to calculate the PDF with PDFgetX3.^136^ Note that scattering profiles of 2D structures with stacking faults can also be calculated with DIFFaX.

### 4.3 EXAFS Spectroscopy

Mn K-edge EXAFS measurements were performed at the ESRF on beamline BM23. Data were collected with a Si 111 monochromated beam in fluorescence-yield mode using a Vortex-ME7 silicon drift detector (Hitachi, Japan). Powders were diluted in cellulose to prevent overabsorption. Four scans of 14 min with a step size of 1.0 eV were recorded and averaged to improve statistics. Data were reduced with the Athena software^137^ and a Labview suite of programs developed at the Advanced Light Source.^138^

### 4.4 TEM

TEM images and SAED patterns were acquired using a 300 kV Themis Z G3 Cs-probe corrected microscope (Thermo Fisher Scientific) equipped of a GATAN 4K OneView camera (resolution ∼ 1.8 Å), and a Super-X windowless emission X-ray spectrometer (EDS).

## Supplementary Material



## Notes

Mn oxidation state from chemical analysis; crystallographic data from XRD simulations; thermogravimetric analyses; TEM images and SAED of δ-MnO_2_ and δ-MnO_2_^HE^-8 at low and high IS; and EXAFS analysis of the Mn_3_ shell (PDF)
