# Supplementary material for: Structure of HEPES-Reduced δ‑MnO2 Nanosheets
Source: Chem Mater. 2026 Apr 15;38(9):4500–16. doi: 10.1021/acs.chemmater.5c03046 (PMC13173508; doi:10.1021/acs.chemmater.5c03046)
Supplement: Supplementary file 1 [file cm5c03046_si_001.pdf]

Structure of HEPES-reduced δ-MnO_2 nanosheets

Alain Manceau[*1,2], Yan Li[*1], Jianlin Liao[1], Valérie Magnin[2], Lorenzo Spadini[3], Catherine Dejoie[1], Olivier Mathon[1], Anne-Claire Gaillot[4]

[1]European Synchrotron Radiation Facility (ESRF), 38043 Grenoble, France
[2]Université Grenoble Alpes, CNRS, ISTerre, 38000 Grenoble, France
[3]Université Grenoble Alpes, CNRS, Institut des Géosciences de l'Environnement, 38000 Grenoble, France
[4]Nantes Université, CNRS, Institut des Matériaux de Nantes Jean Rouxel, IMN, 44000 Nantes, France

Supplementary Tables

Table S1. Manganese oxidation state

Sample	Mn(II)	SD	Mn(III)	SD	Mn(IV)	SD	AMOS	SD
Low IS δ-MnO_2-6	0.01	0.00	0.11	0.00	0.88	0.00	3.87	0.00
Low IS δ-MnO_2-8	0.01	0.01	0.11	0.01	0.88	0.01	3.88	0.00
Low IS δ-MnO_2^{HE}-6	0.06	0.00	0.29	0.01	0.65	0.00	3.59	0.01
Low IS δ-MnO_2^{HE}-8	0.04	0.00	0.26	0.00	0.70	0.01	3.66	0.00
High IS δ-MnO_2-6	0.01	0.00	0.09	0.00	0.90	0.00	3.89	0.00
High IS δ-MnO_2-8	0.01	0.00	0.07	0.00	0.92	0.00	3.92	0.00
High IS δ-MnO_2^{HE}-6	0.06	0.00	0.29	0.00	0.65	0.00	3.59	0.01
High IS δ-MnO_2^{HE}-8	0.03	0.00	0.22	0.01	0.74	0.01	3.71	0.01

Table S2. Atomic coordinates of the Mn and O atoms represented with the $P\bar{3}m1$ space group without *c* translation

	x	*y*	*z*	*B* (Å^2)
Mn_L	0	0	0.0	0.5
O_L	2/3	1/3	0.129	1.0
Mn_{IL}	0	0	0.280	1.0
H_2O^b	1/3	2/3	0.480	2.0
Na	0.950	0.475	1/2	2.0
H_2O^s	0.400	0.200	1/2	2.0

Equivalent positions: (-*x*+*y*,-x,*z*) and (-*y*,*x*-*y*,*z*). All positions were fixed to previous values (Manceau et al., J. Appl. Cryst., 2013, 46, 193-209).

Table S3. Unit-cell parameters, atom-independent parameters, and site occupancies determined by Bragg rod simulation of the X-ray scattering data.

Sample	a=b (Å)	CSD (Å)[1]	σ[ln(CSD)][2]	δ[3]	Mn_L	O_L	Mn_{IL}	H_2O^b	Na	H_2O^s
Low IS δ-MnO_2-6	2.830	50	0.50	0.00	0.90	2.00	0.08	0.24	0.21	0.46
Low IS δ-MnO_2-8	2.830	50	0.50	0.00	0.85	2.00	0.12	0.33	0.29	0.40
Low IS δ-MnO_2^{HE}-6	2.834	65	0.40	0.20	0.77	2.00	0.23	0.69	0.03	0.00
Low IS δ-MnO_2^{HE}-8	2.834	65	0.40	0.20	0.77	2.00	0.23	0.69	0.05	0.00

[1]Diameter of the disk-shaped domains in the *ab* plane. [2]Log-normal distribution. [3]Strain parameter (Manceau et al., 2013).

Supplementary Figures

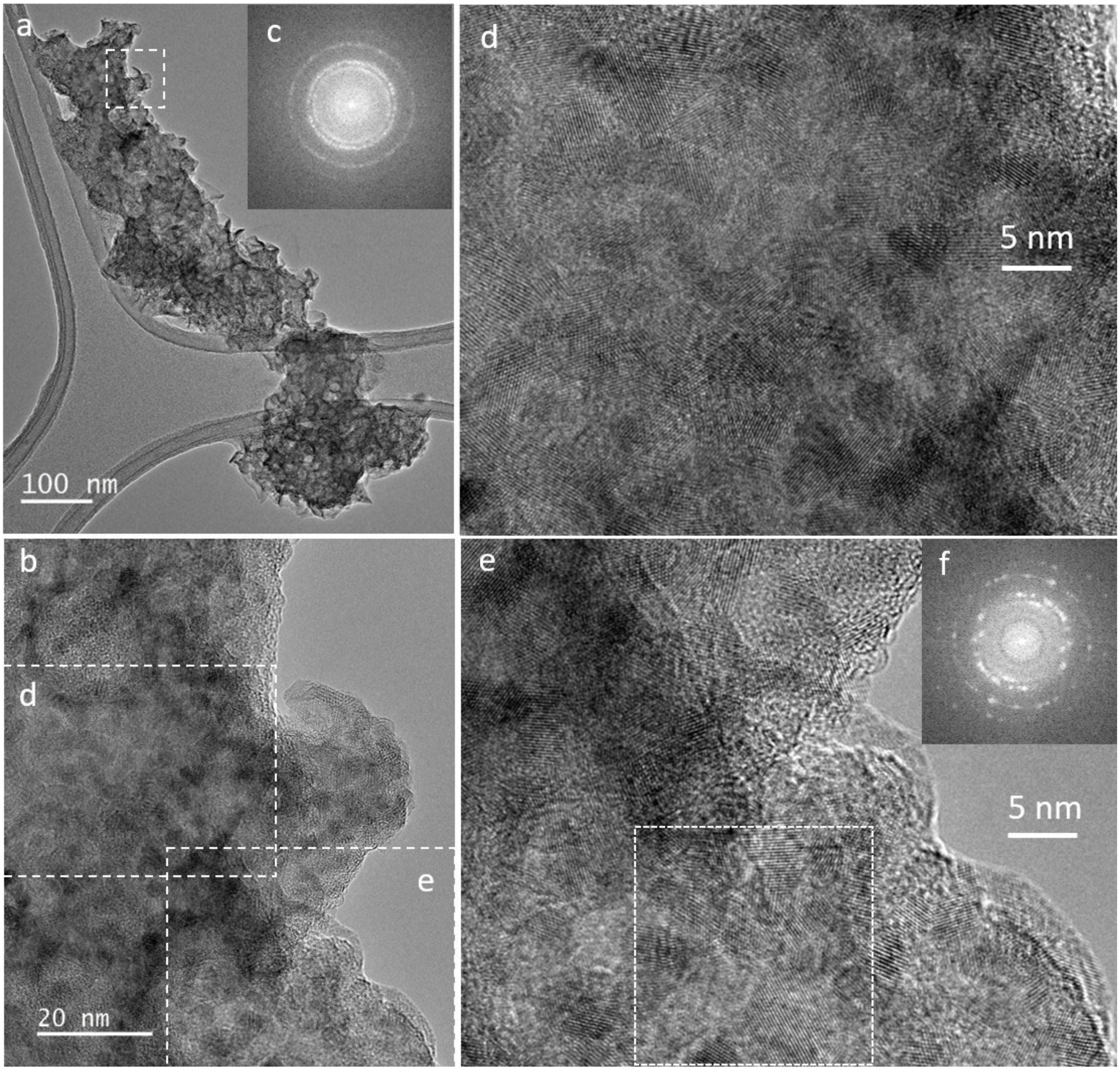

Figure S1. Transmission electron images of δ-MnO_2. (a) Full aggregate. (b) HRTEM image of the framed area in (a). (c) FFT pattern of (b) showing the presence of crystalline nanodomains of hexagonal layer symmetry in the *ab* plane. (d,e) High magnification of the framed area in (b) showing 5 nm wide nanodomains. (f) FFT of the framed area in (e) showing that the nanodomains are single crystals.

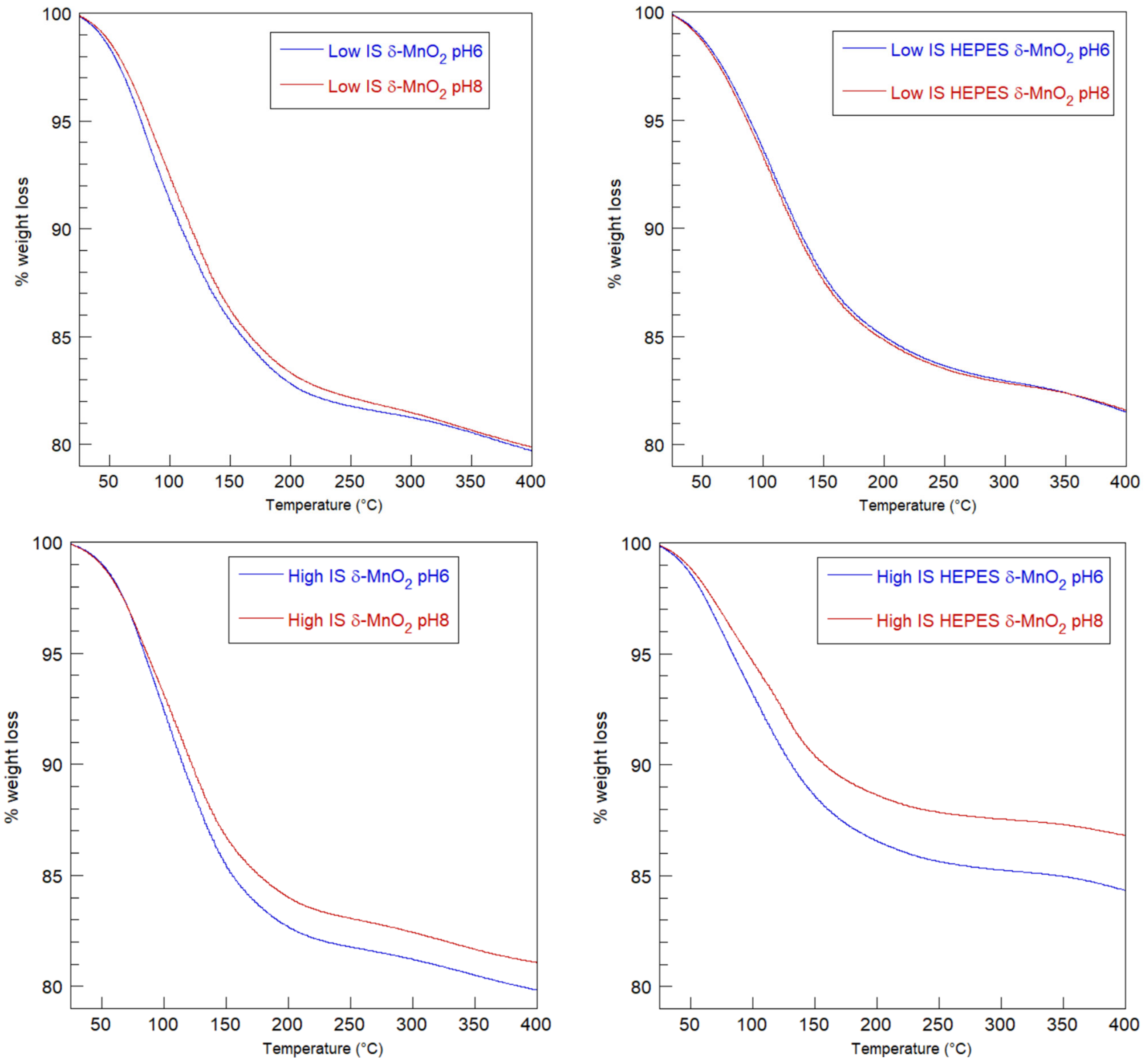

Figure S2. Weight loss normalized to sample weight at room temperature measured by thermogravimetric analysis.

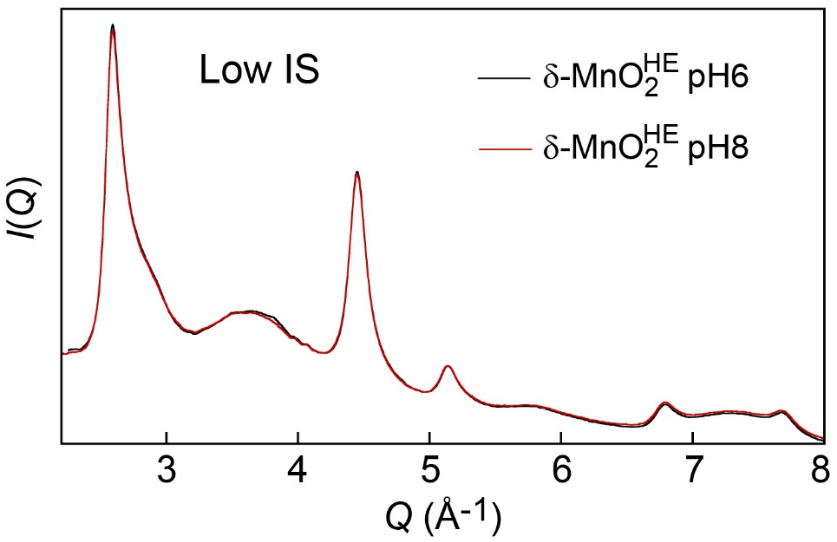

Figure S3. High-energy diffractograms of δ-MnO_2^{HE} at pH 6 and 8 and low ionic strength (< 0.01 M NaCl).

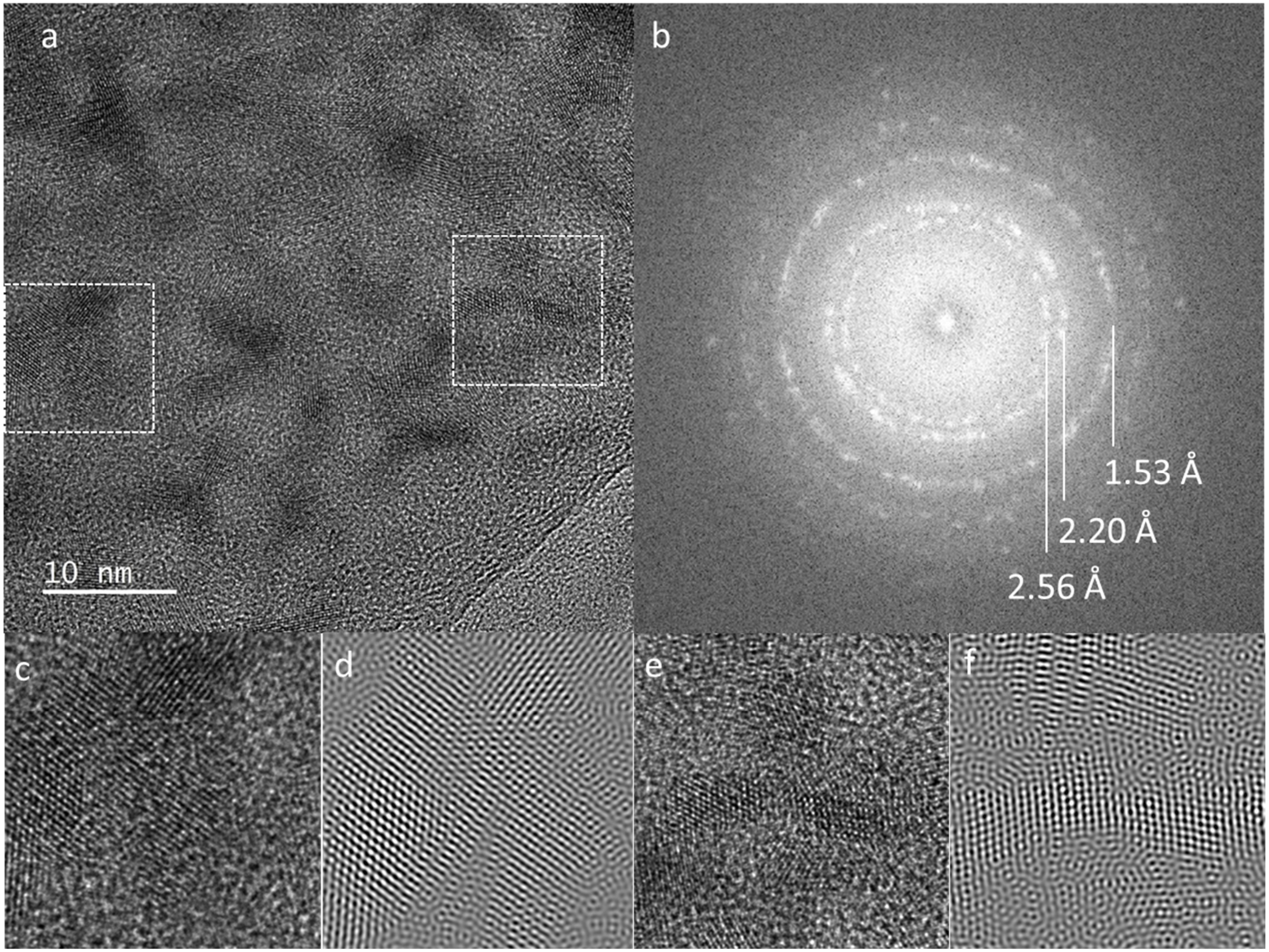

Figure S4. (a) HRTEM image of low-IS δ-MnO_2^{HE}-8 nanocrystals in an amorphous matrix. (b) FFT of image (a). (c,e) Magnified area of the ~3-6 nm nanocrystals framed in (a). (d,f) Filtered images of (c,e) corresponding to the second and first ring of frequencies of the FFT (around 2.20 Å and 1.53 Å), respectively.

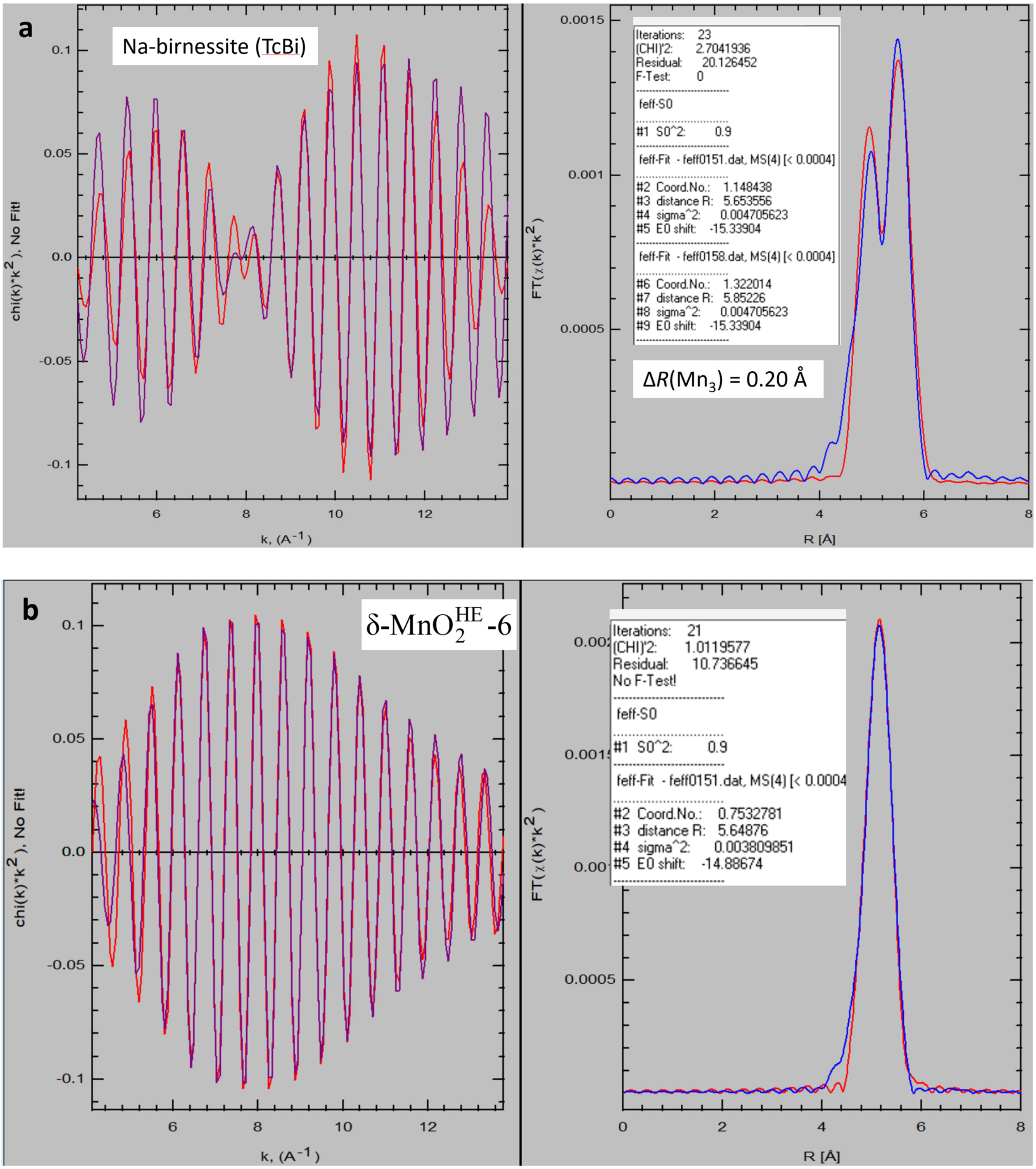
a
Na-birnessite (TcBi)
chi(k)*k^2), No Fit!
k, (A^-1)
FT(χ(k)*k^2)
R [Å]
Iterations: 23
(CHI)^2: 2.7041936
Residual: 20.126452
F-Test: 0
feff-S0
#1 S0^2: 0.9
feff-Fit - feff0151.dat, MS(4) [< 0.0004]
#2 Coord.No.: 1.148438
#3 distance R: 5.653556
#4 sigma^2: 0.004705623
#5 E0 shift: -15.33904
feff-Fit - feff0158.dat, MS(4) [< 0.0004]
#6 Coord.No.: 1.322014
#7 distance R: 5.85226
#8 sigma^2: 0.004705623
#9 E0 shift: -15.33904
ΔR(Mn3) = 0.20 Å
b
δ-MnO2HE-6
Iterations: 21
(CHI)^2: 1.0119577
Residual: 10.736645
No F-Test!
feff-S0
#1 S0^2: 0.9
feff-Fit - feff0151.dat, MS(4) [< 0.0004
#2 Coord.No.: 0.7532781
#3 distance R: 5.64876
#4 sigma^2: 0.003809851
#5 E0 shift: -14.88674

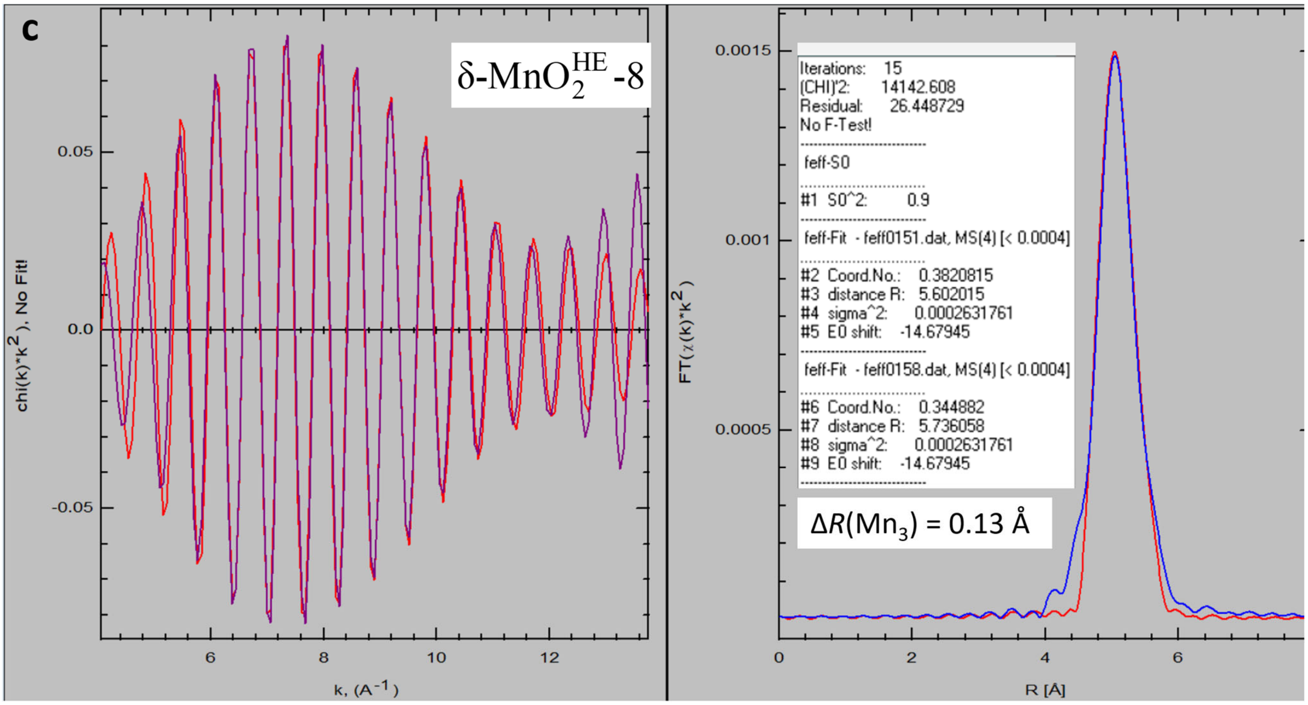

Figure S5. EXAFS fit of the Mn3 shell for Na-birnessite (TcBi) (a), δ-MnO_2^{HE}-6 (b), and δ-MnO_2^{HE}-8 (c).

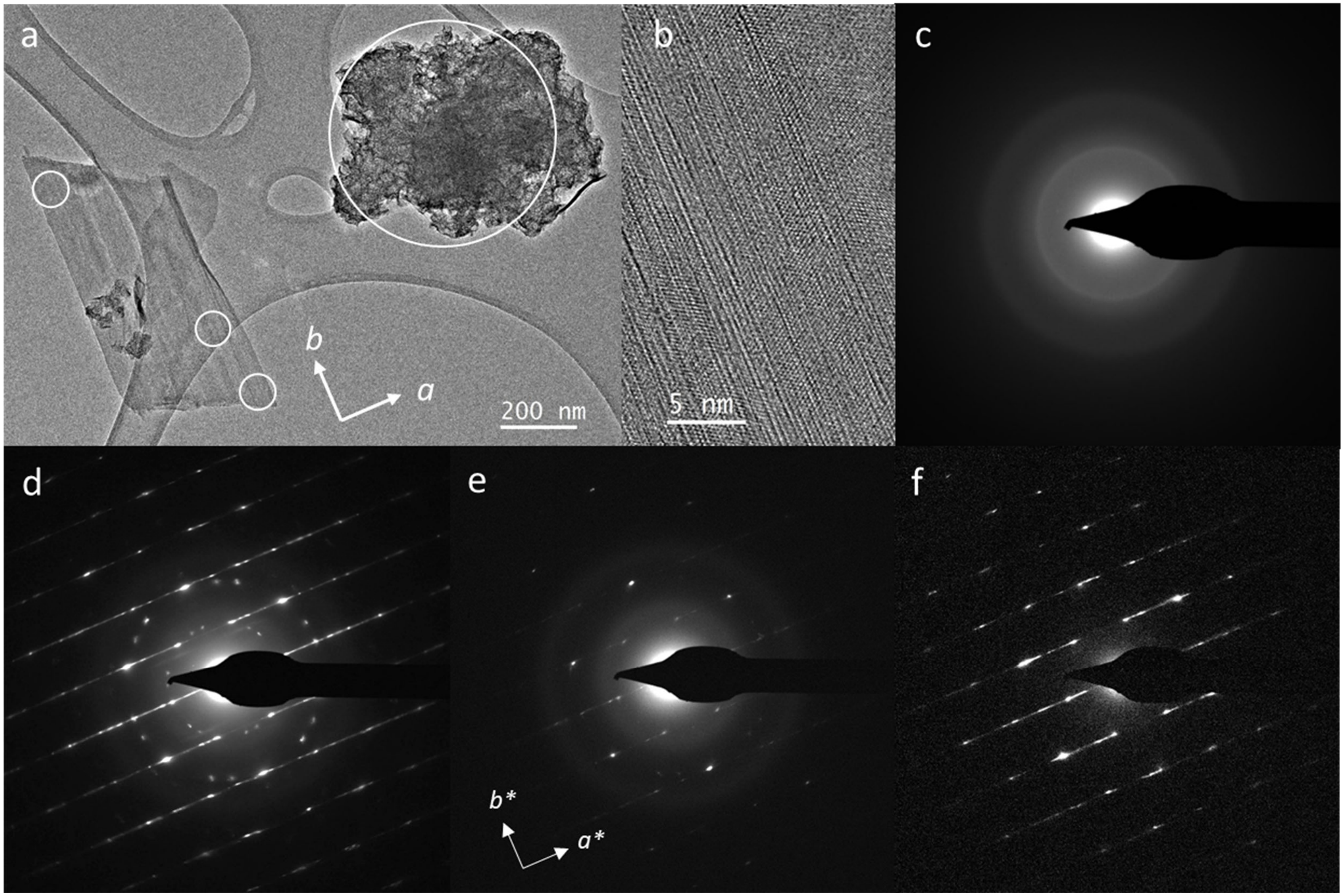

Figure S6. Transmission electron images and SAED patterns of high-IS δ-MnO_2^{HE}-8. (a) TEM image of a large and three small flake aggregates and a lath-shaped crystal. (b) HRTEM of the lath-shaped crystal in (a), showing irregular intensity contrast in the *a* direction perpendicular to the *b* elongation axis of the lath. (c) SAED pattern of the large poorly crystalline aggregate in (a). (d-f) SAED patterns of the three circled areas in (a), showing superlattice reflections and streaking along the *a** direction due to the irregular structure ordering in the *a* direction.

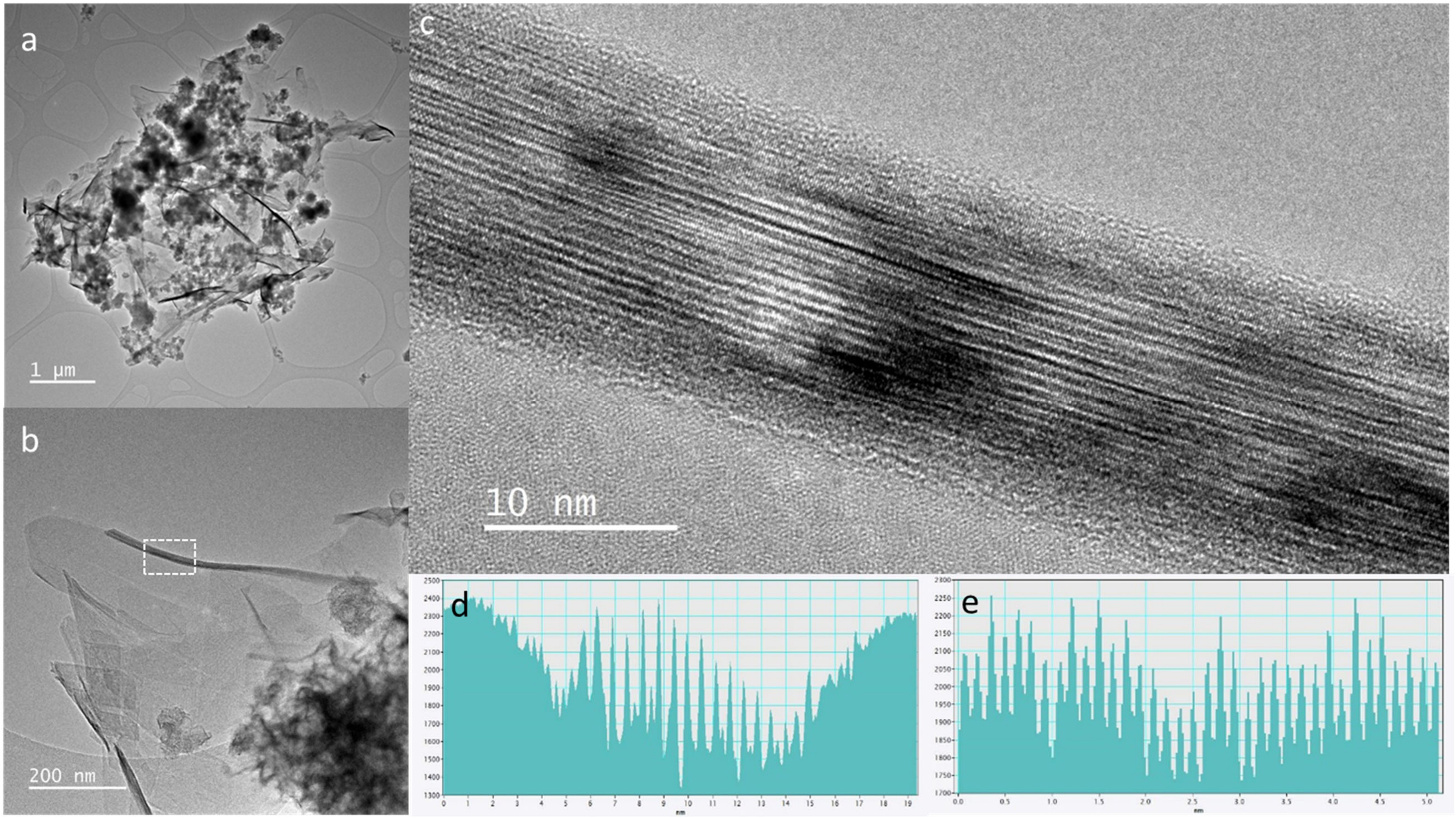

Figure S7. Transmission electron images of high-IS δ-MnO_2^{HE}-8. (a) TEM image of an aggregate of flakes and crystals with rolled edges. (b) Enlarged TEM image of a platelet with layers rolled on the edges along the *b* direction. (c) HRTEM image of the rolled layers framed in (b). The δ-MnO_2 layers are viewed perpendicularly to the *ab* plane ([001] zone axis). The crystal contains approximately 20 δ-MnO_2 layers, explaining the intense 001 XRD reflection of high-IS δ-MnO_2^{HE}-8 (Figure 5b). (d-e) Intensity profiles along (1.57 Å) and perpendicular to the well-stacked layers (5.6 Å *d*(001) spacing).

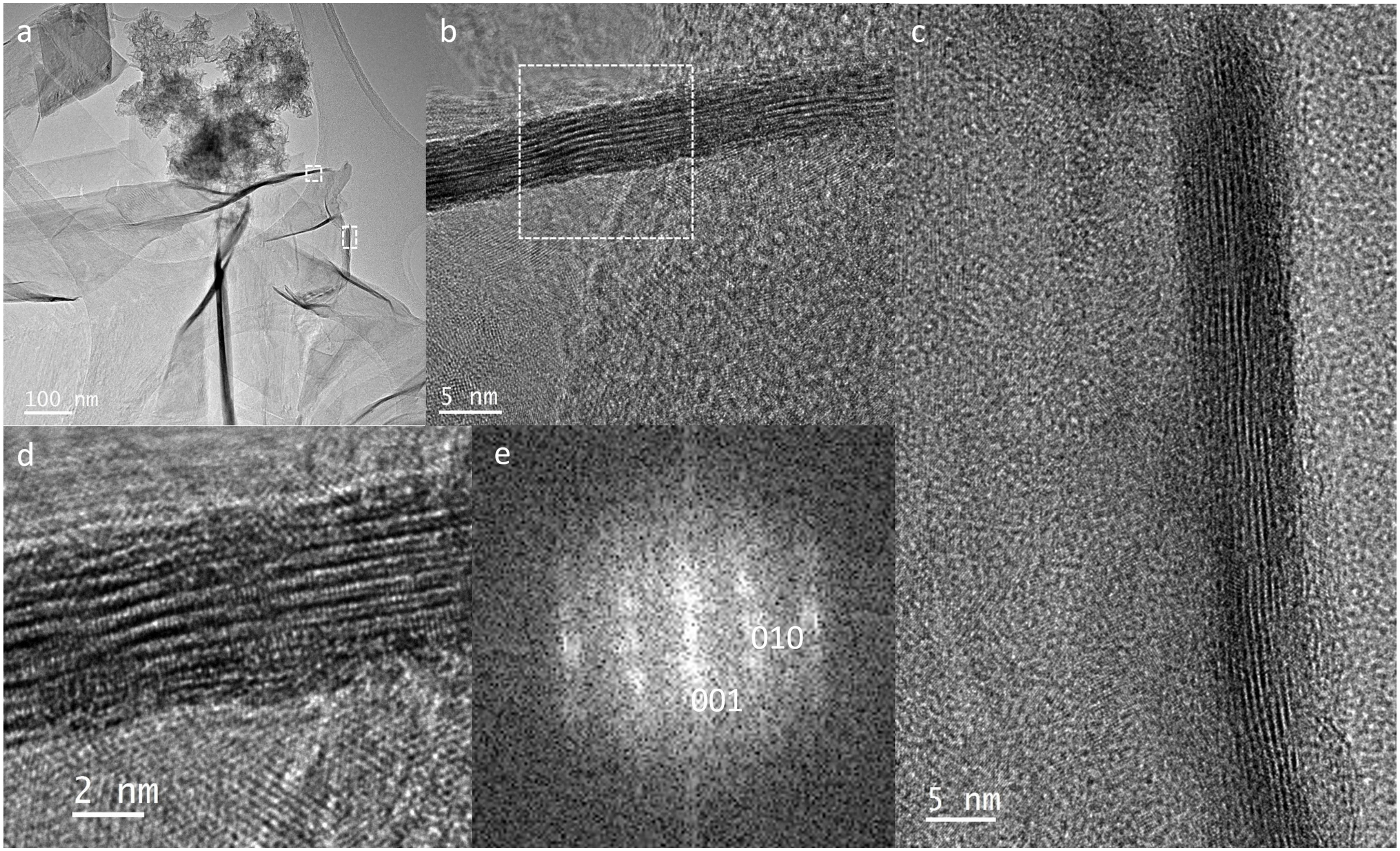

Figure S8. (a) TEM image of large flakes with rolled edges and small flakes aggregates for high-IS δ-MnO_2^{HE}-8. (b,c) HRTEM images of the rolled dehydrated δ-MnO_2 layers framed in (a) viewed along the [100] direction. (d) Enlarged view of the layers framed in (b). The layer planes ondulate with a 5-7 nm periodicity. (e) FFT of the HRTEM image in (d), showing a periodicity of 2.85 Å and 5.30 Å along the *b* and *c* directions, respectively.

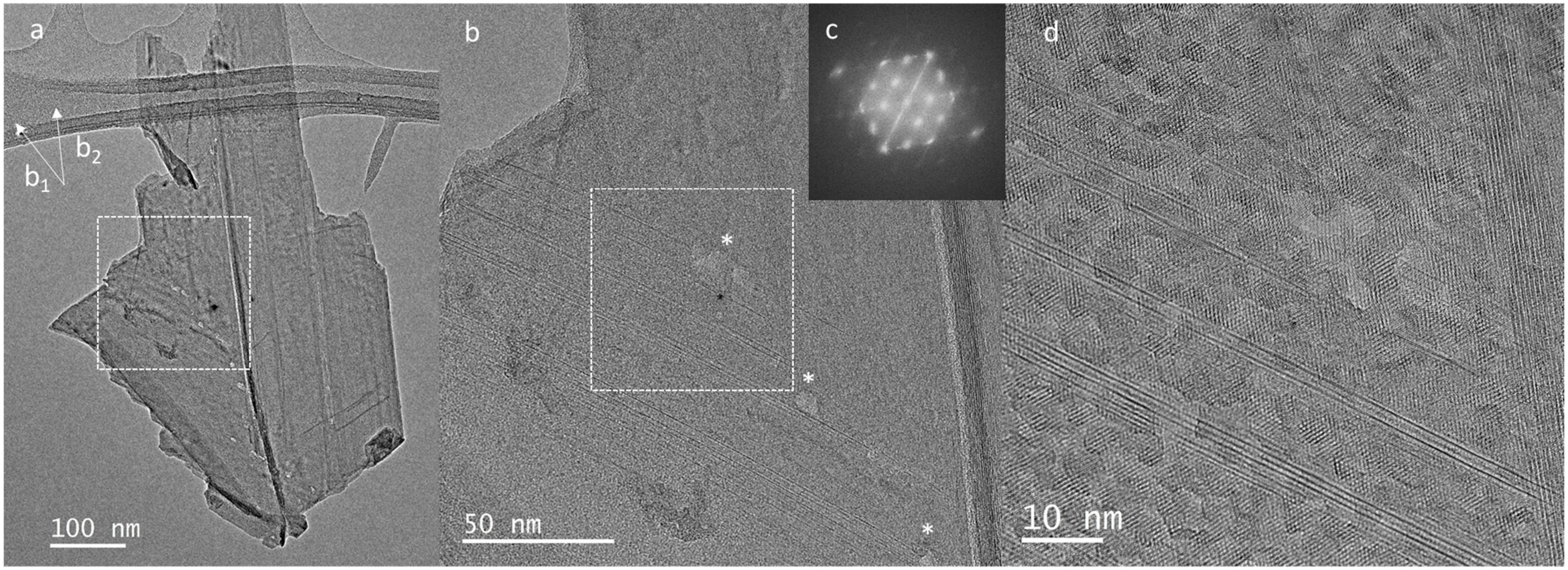

Figure S9. (a) TEM image of a twinned crystal of high-IS δ-MnO_2^{HE}-8 elongated along the b_1 and b_2 directions. (b) Enlarged image of the twinning boundary, showing defects and interrupted layers (star symbols). (c) FFT of the left twin, showing streaking due to the irregular lattice superperiodicity perpendicularly to the b_1 direction. (d) HRTEM image of the framed area in (b), showing an alternation of well-ordered regions with superlattice fringes parallel to the b_1 elongation axis, likely caused by local ordering of interlayer Na and layer Mn(III) cations, along with more defective and beam-sensitive hexagonal nanodomains. The well-ordered domains develop through the oriented growth of the hexagonal domains.

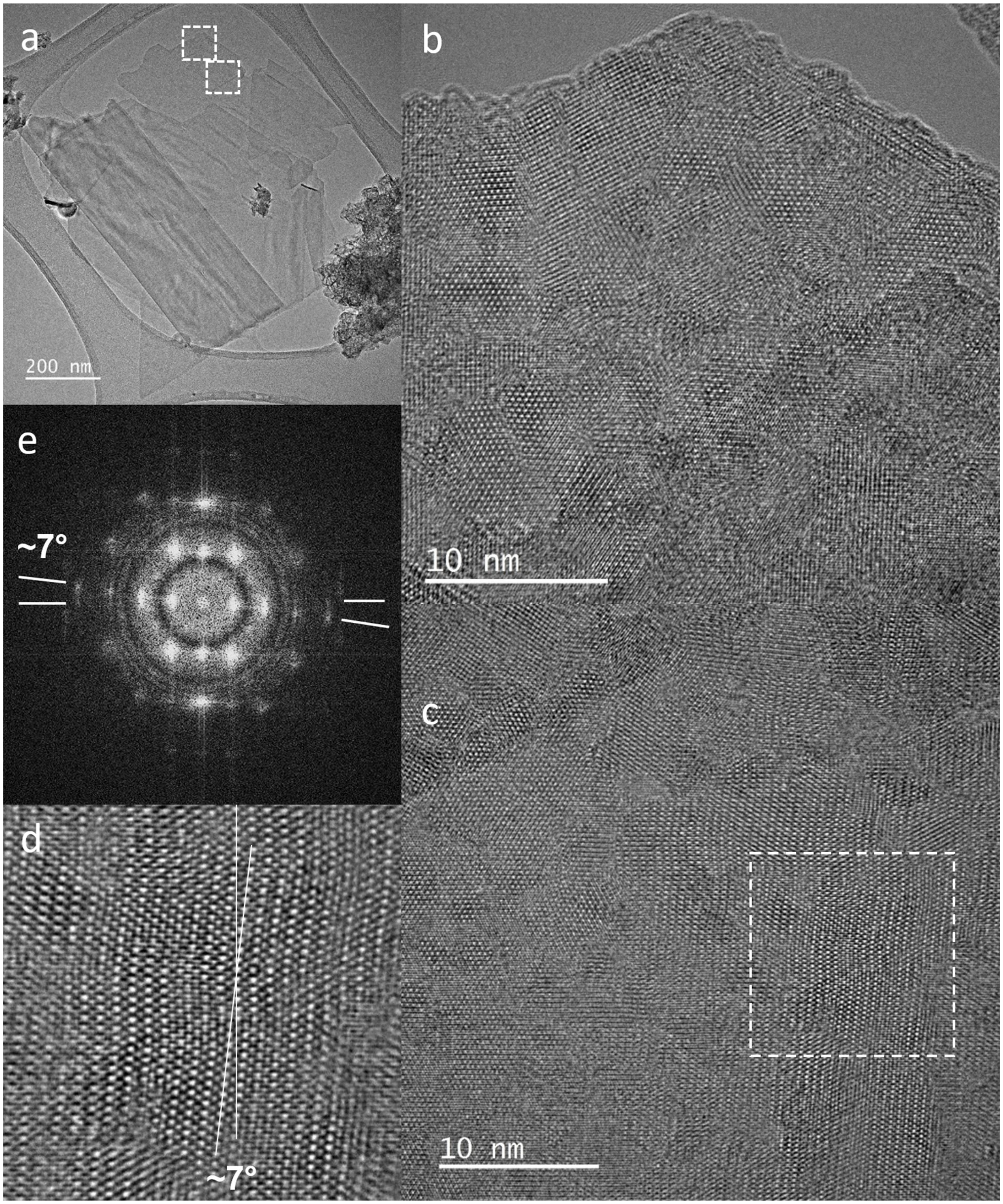

Figure S10: (a) TEM image of high-IS δ-MnO_2^{HE}-8 flakes of different crystallinity. The upper particle has less-defined edges than the lower one. (b,c) HRTEM images of the upper particle, consisting of an aggregation of nanocrystals. (d) Enlargement of the framed area in (c), showing a local distortion of the lattice. The white fringe maxima follow ondulating lines that deviate by ~7° from the main lattice. The undulating pattern is attributed to variable distributions of the Mn(III) cations and orientations of the elongated Jahn-Teller axes of the Mn(III) octahedra within the MnO_2 layer, resulting from the merging of the nanocrystalline domains (visible in some areas) during the crystal growth. (e) FFT pattern of (d). The alternation of intense and weaker fringe lines in (d) appears as a loss of cell centering in the FFT.

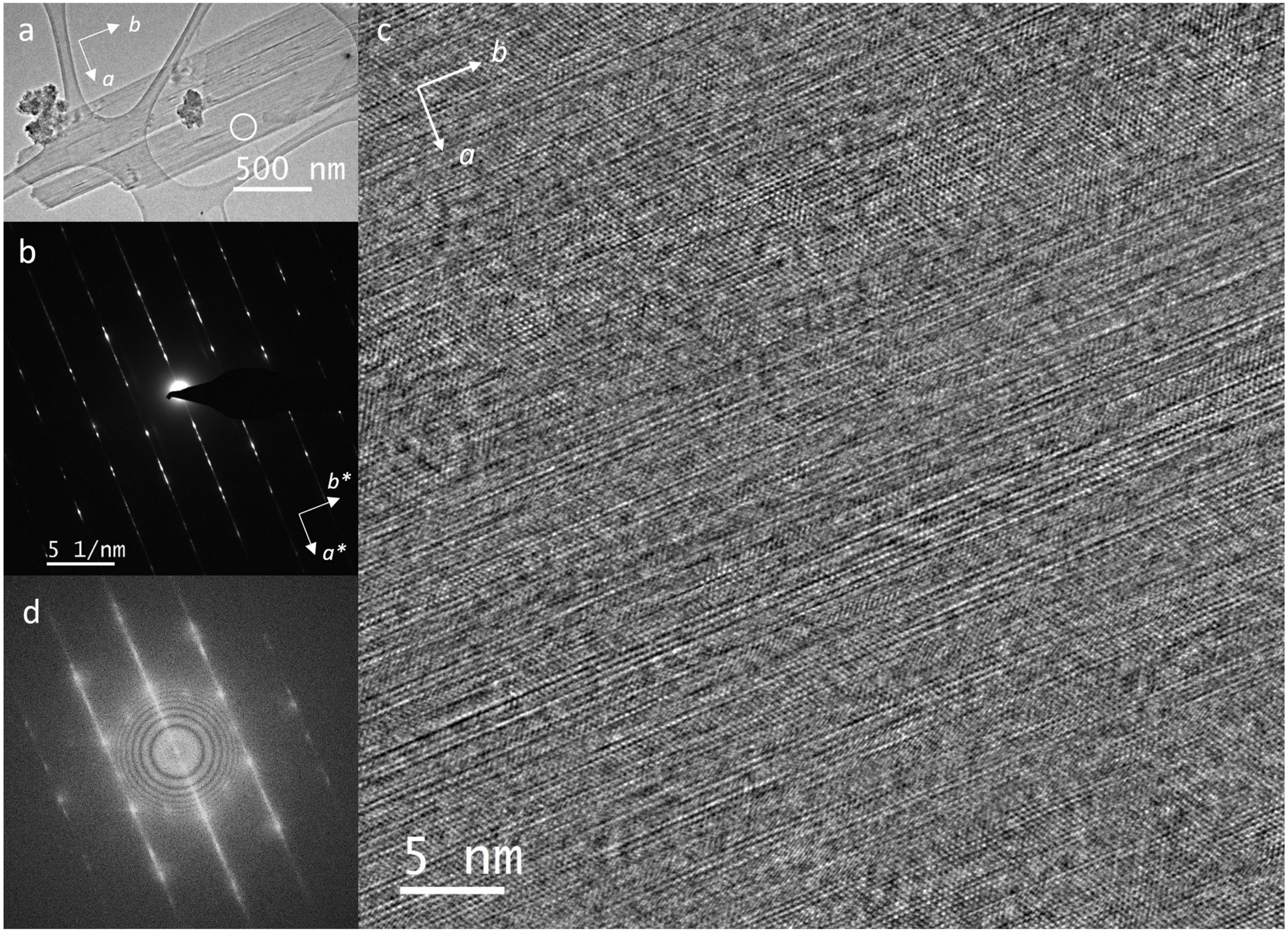

Figure S11. (a) TEM image of a high-IS δ-MnO_2^{HE}-8 lath elongated along the *b* direction ([001] axis). (b) SAED pattern of the circled area in (a) with incommensurate superlattice spots and streaking along the a* direction. (c) HRTEM image of the circled area in (a). (d) FFT pattern of the (c) image. The streaking observed in the SAED and FFT patterns arises from structural disorder along *a* in (c).

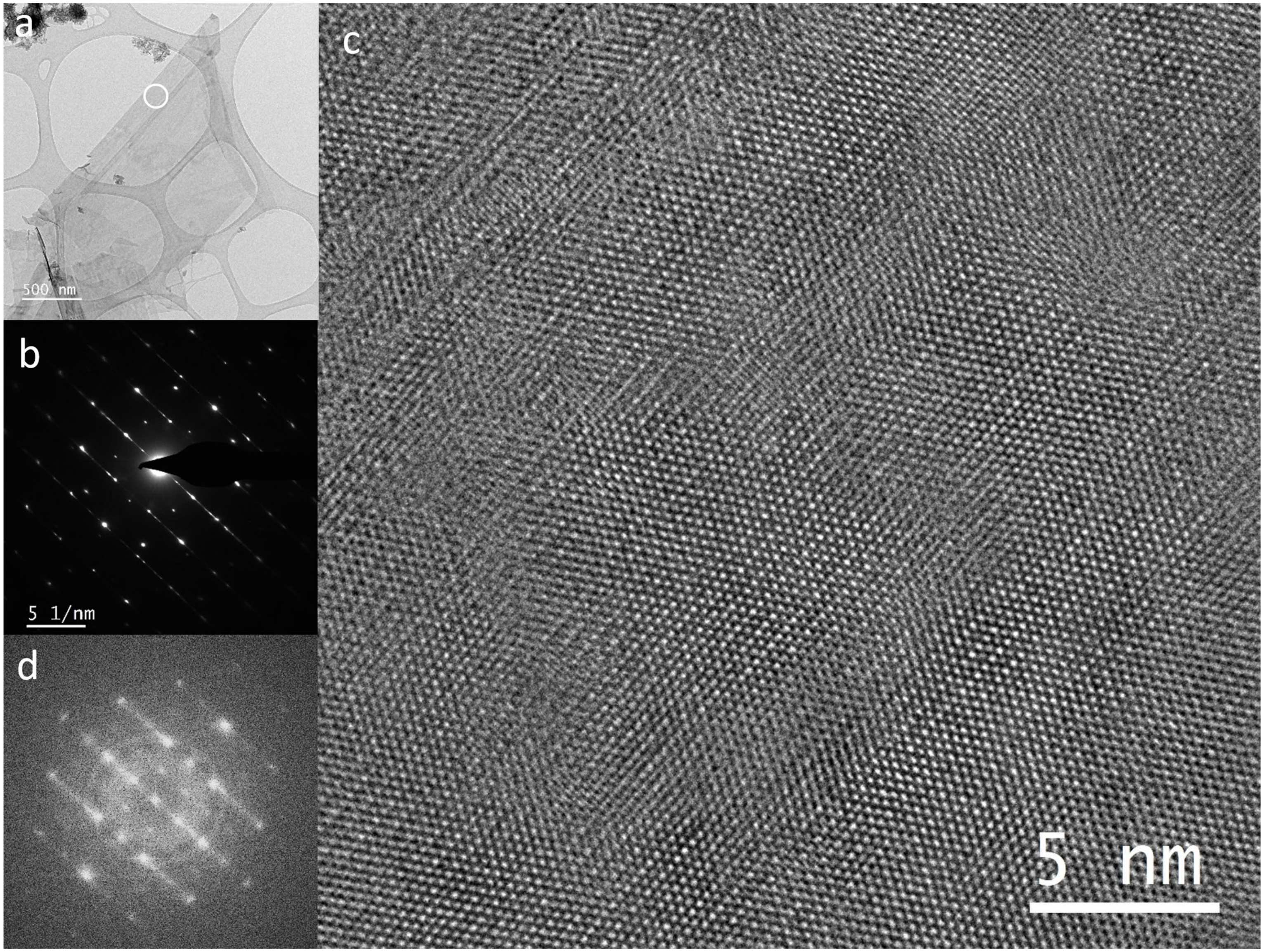

Figure S12. (a) TEM image of a high-IS δ-MnO_2^{HE}-8 lath elongated along the *b* direction ([001] axis). (b) SAED pattern of the circled area in (a) with incommensurate superlattice spots and streaking along the a* direction. (c) HRTEM image of the circled area in (a), showing large defectless regions. Some remnants of the original 7-nm nanodomains appear to persist. (d) FFT pattern from HRTEM image in (c).

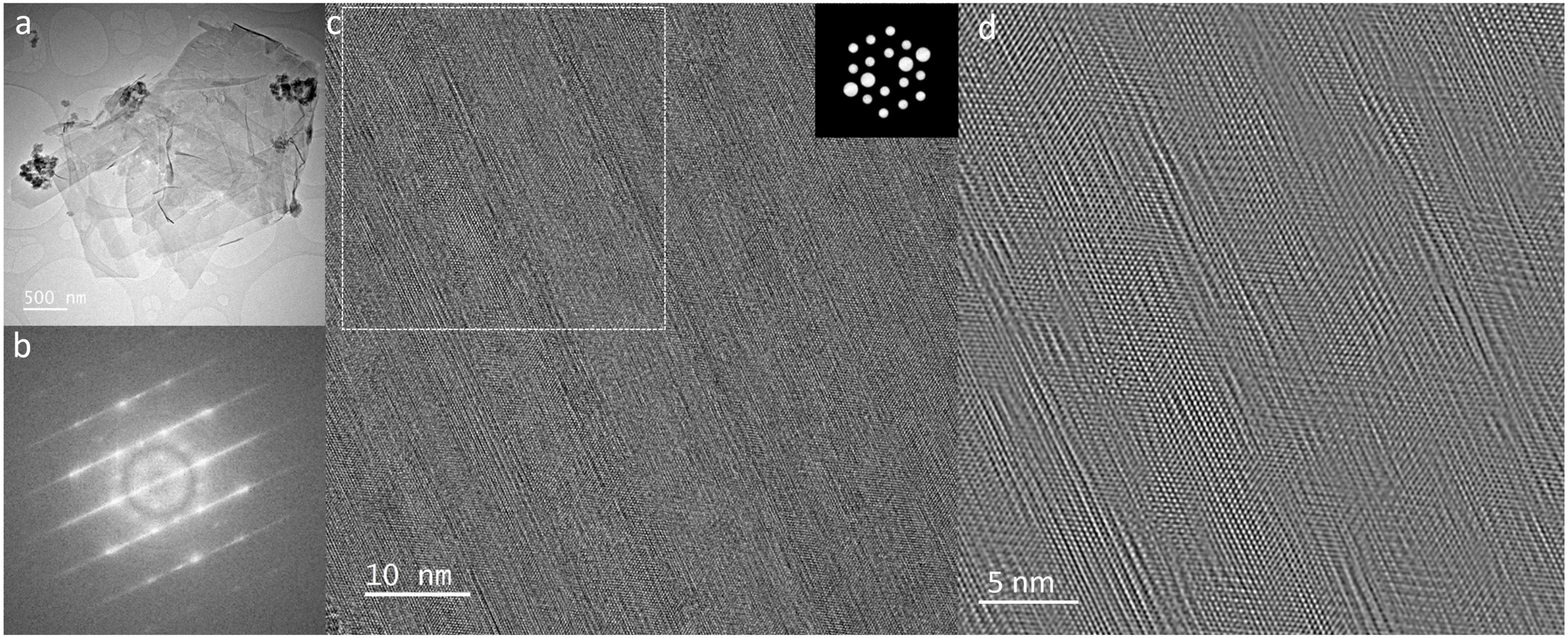

Figure S13. (a) TEM image of large platelets and small flake aggregates for high-IS δ-MnO_2^{HE} -8 . (b,c) HRTEM image and FFT from the lower left lath in (a). (d) Filtered image of the framed area in (b), displaying the 2D periodic arrangement of the Na and Mn(III) cations and the lattice distortion due to the presence of Jahn-Teller Mn(III) cations within the δ-MnO_2 layers

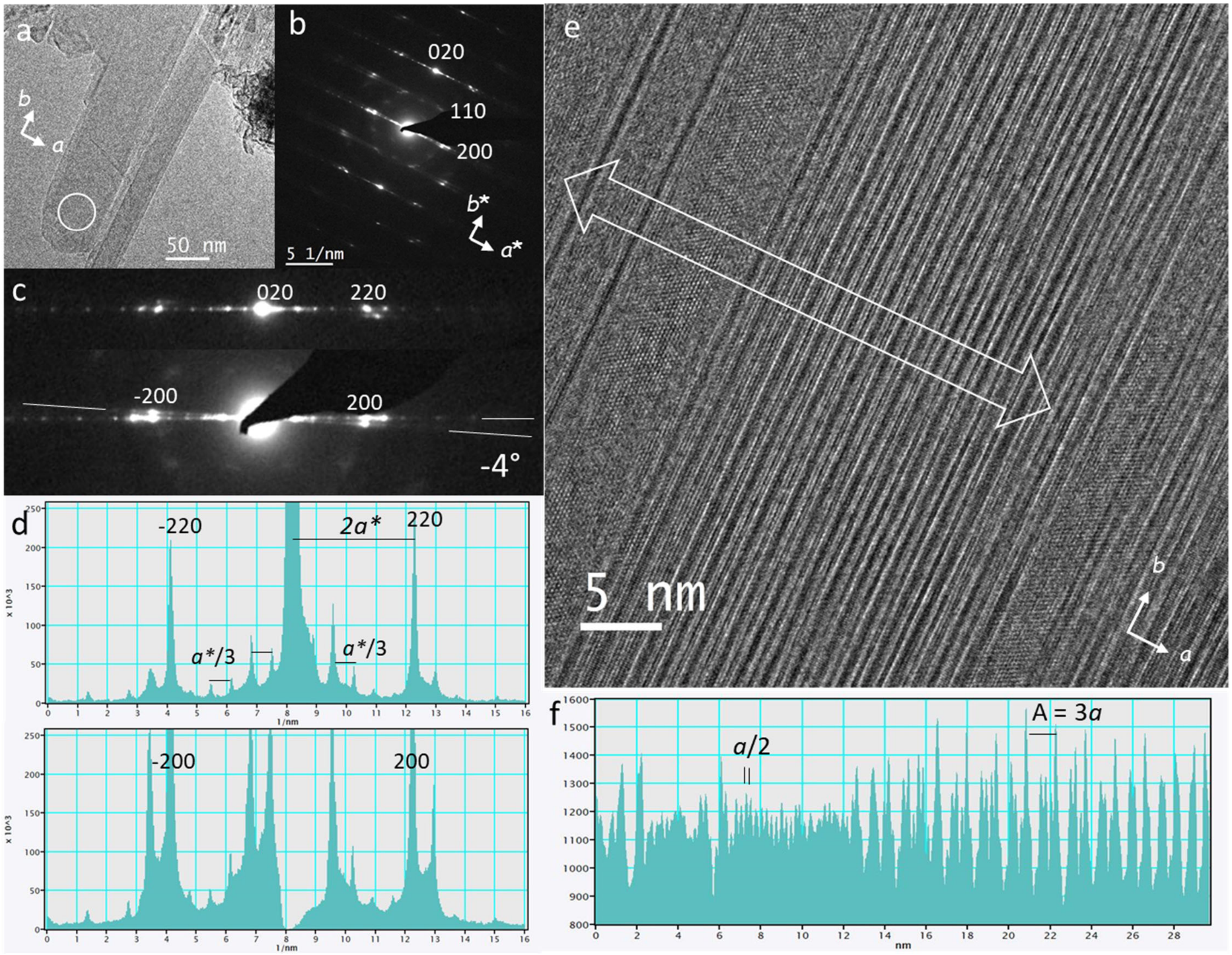

Figure S14. (a) TEM image of a high-IS δ-MnO_2^{HE}-8 lath. (b) SAED pattern of the circled area in (a). (c-d) Zoom on the *h*20 and *h*00 reflections lines and corresponding intensity profiles through the *hk*0 reflections, showing a clear 2*a**/6 superlattice reflections (corresponding to a *A* = 3*a* non-centered supercell). Additional reflections appear on a line passing through the center of the pattern and tilted by about 4°. (e) HRTEM image of the same region in the lath, showing *a*/2 and 3*a* periodicities. (f) Intensity profile through the HRTEM image (double arrow).

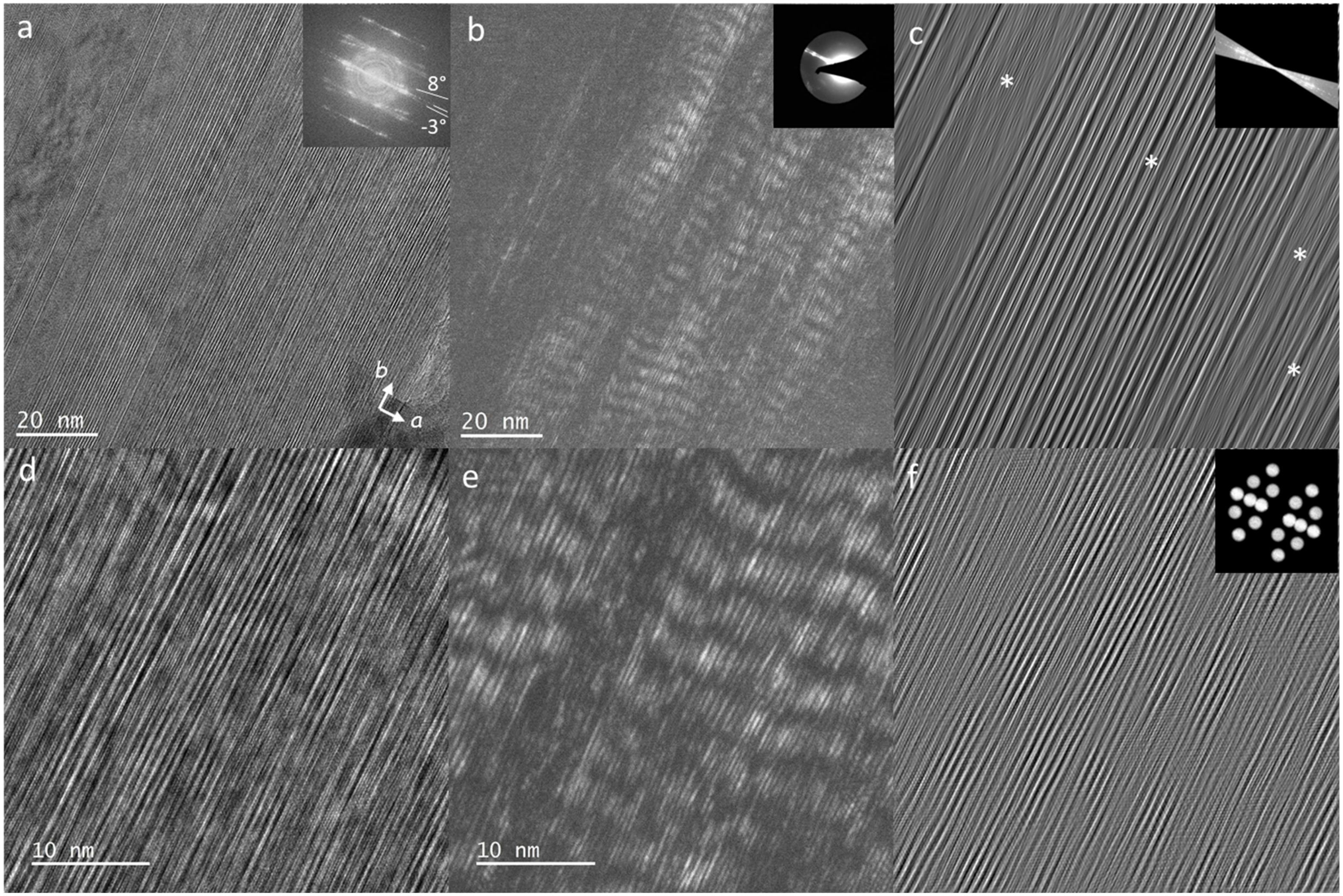

Figure S15. HRTEM images of the high-IS δ-MnO_2^{HE}-8 lath in Figure S14a. (a) Large view and corresponding FFT pattern in inset, showing additional super-reflection spots on lines tilted by 8° and -3° with respect to the main lattice (as in Figure 14c). (b) Dark-field HRTEM image of the same area as (a), obtained using an objective aperture of 20 µm centered on the super-reflections. The dark-field image confirms the correspondence between the super-reflection spots and the brighter lines in (a), and reveals modulations in cation ordering along the *b* direction.

(c) Filtered image of an enlarged area in (a), using a wedge mask (in inset) to select all super-reflection spots, including those on lines tilted by 8° and -3°. The fringes are not all straight but appear wavy in some places (star symbols). This wavy structure explains the tilt of the surstructures on the FFT. (d) HRTEM image of an enlarged zone in (a). (e) Dark-field HRTEM obtained with the same aperture as (b), showing the loss of the superstructure contrast in the *b* direction every 5 nm, and a wavy alignment of the ordered domains in the *a* direction. The zebra-shaped pattern could result from an order-disorder pattern of the Na and associated Mn(III) cations or from a corrugating effect of the MnO_2 layers caused by structural accommodation induced by the distorted Mn(III) octahedra. (f) Filtered image with a spot mask (in inset) revealing the overall waving alignment of the Mn lattice along the *b*-direction.

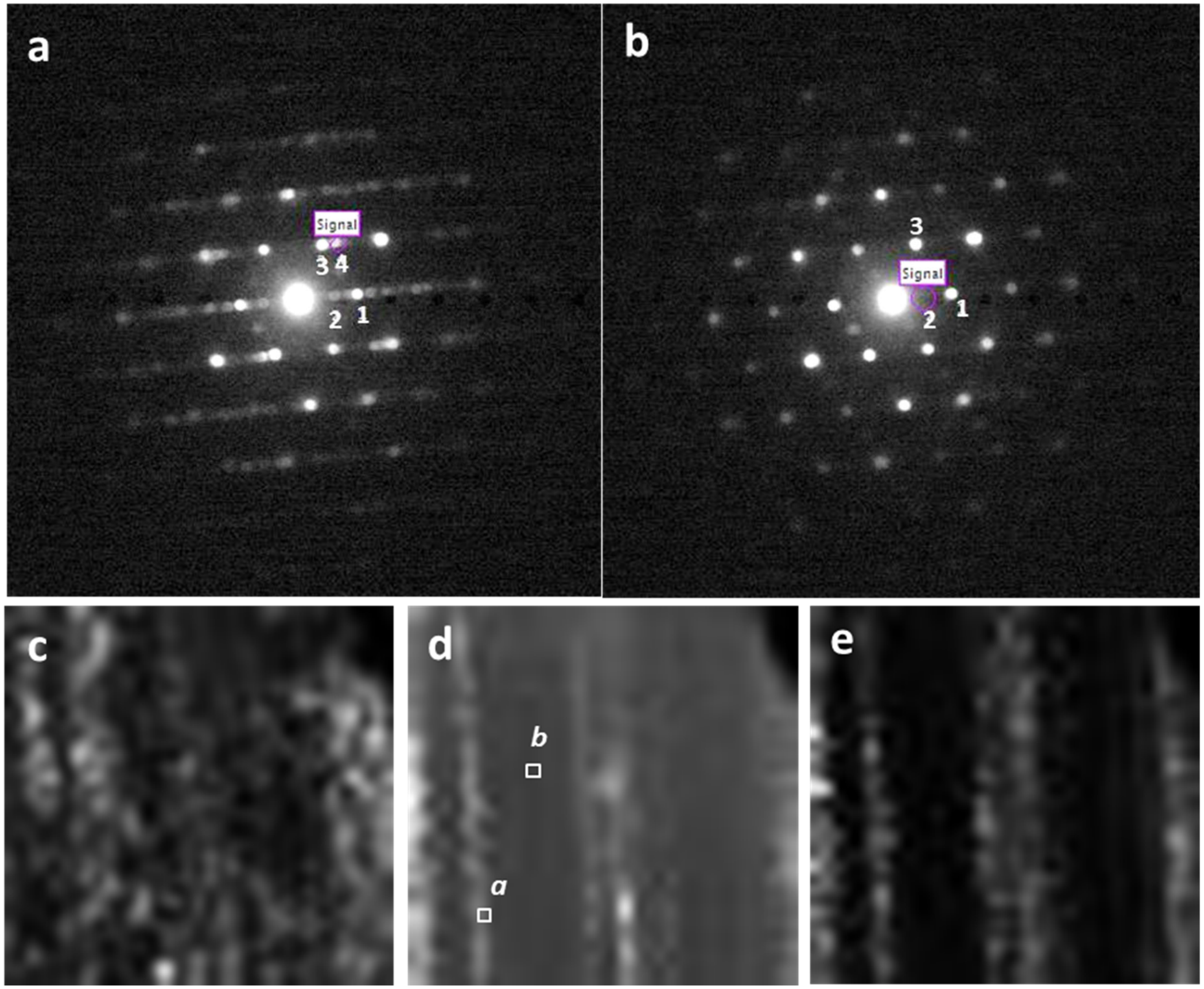

Figure S16. Electron diffraction mapping using 4D-STEM from the GATAN GMS suite, illustrating the local variation in the Na and Mn(III) ordering. (a-b) Two individual micro electron diffraction patterns showing the presence or absence of A = 3a super-reflections (beam positions are indicated in d). (c) Virtual dark-field image reconstructed using the main lattice 200 and 110 reflections labelled 1 and 3 in (a) and (b). (d-e) Virtual dark-field image reconstructed using the super-reflections labelled 2 in (a) and (b) and 4 in (a). The super-reflection 4 is absent from (b).
